# Deuteration as a General Strategy to Enhance Azobenzene-Based Photopharmacology

**DOI:** 10.1002/anie.202408300

**Published:** 2024-08-19

**Authors:** Kilian Roßmann, Alberto J. Gonzalez-Hernandez, Rahul Bhuyan, Caspar Schattenberg, Han Sun, Karl Börjesson, Joshua Levitz, Johannes Broichhagen

**Affiliations:** Leibniz-Forschungsinstitut für Molekulare Pharmakologie (FMP), 13125 Berlin, Germany; Department of Biochemistry, Weill Cornell Medicine, New York, NY 10065, USA; Department of Chemistry and Molecular Biology, University of Gothenburg, 413 90 Gothenburg, Sweden; Leibniz-Forschungsinstitut für Molekulare Pharmakologie (FMP), 13125 Berlin, Germany; Leibniz-Forschungsinstitut für Molekulare Pharmakologie (FMP), 13125 Berlin, Germany; Department of Chemistry and Molecular Biology, University of Gothenburg, 413 90 Gothenburg, Sweden; Department of Biochemistry, Weill Cornell Medicine, New York, NY 10065, USA; Leibniz-Forschungsinstitut für Molekulare Pharmakologie (FMP), 13125 Berlin, Germany

**Keywords:** Photopharmacology, Azobenzene, Deuteration, Ion Channel, G Protein-Coupled Receptor

## Abstract

Chemical photoswitches have become a widely used approach for the remote control of biological functions with spatiotemporal precision. Several molecular scaffolds have been implemented to improve photoswitch characteristics, ranging from the nature of the photoswitch itself (e.g. azobenzenes, dithienylethenes, hemithioindigo) to fine-tuning of aromatic units and substituents. Herein, we present deuterated azobenzene photoswitches as a general means of enhancing the performance of photopharmacological molecules. Deuteration can improve azobenzene performance in terms of light sensitivity (higher molar extinction coefficient), photoswitch efficiency (higher photoisomerization quantum yield), and photoswitch kinetics (faster macroscopic rate of photoisomerization) with minimal alteration to the underlying structure of the photopharmacological ligand. We report synthesized deuterated azobenzene-based ligands for the optimized optical control of ion channel and G protein-coupled receptor (GPCR) function in live cells, setting the stage for the straightforward, widespread adoption of this approach.

## Introduction

Photopharmacology represents a powerful means of optically controlling biological function through the use of lightsensitive compounds.^[1,2]^ In addition to its use in a wide variety of basic science applications,^[3-12]^ photopharmacology has now entered clinical trials via KIO-301, a photoswitchable ion channel blocker with great potential for vision restoration.^[13]^ This compound utilizes an azobenzene-based photoswitch, which represents one of the primary chemical moieties used in photopharmacological probes.^[14]^ Despite their many advantageous properties,^[15]^ azobenzene photoswitch performance is typically limited by light sensitivity (i.e. molar extinction coefficient), photoisomerization efficiency (i.e. photoisomerization quantum yields), and macroscopic photoswitching speed which together reduce their ability to enable robust and rapid light-dependent control in complex biological systems. In recent years, multiple strategies have emerged to improve the properties of azobenzene-based photoswitches. Most typical has been derivatization of the azobenzene itself, which can enhance critical photophysical properties (extinction coefficient, wavelength tuning, bistability etc.) by a diverse array of chemical modifications,^[14]^ introducing heterocycles^[16,17]^ or halogen atoms^[18,19]^ on the aromatic units, creating red-shifted systems,^[20-23]^ reversing isomerization wavelengths (UV to *trans*; green light to *cis*)^[24]^ the installation of sensitive “antennas” for 2-photon activation^[25]^ or the quest for faster switches.^[23,26,27]^ The latter has frequently been noted as a need in studies which apply photopharmacology to neuronal receptors and ion channels.^[28-31]^ To enable improved target selectivity and genetic precision, covalent tethering to cysteines or self-labelling enzymes (e.g. SNAP-tag) has also been used. We recently reported a strategy to effectively improve tethered photopharmacology efficiency by branching multiple azobenzene switches onto the same molecule.^[32,33]^

**Figure 1.**
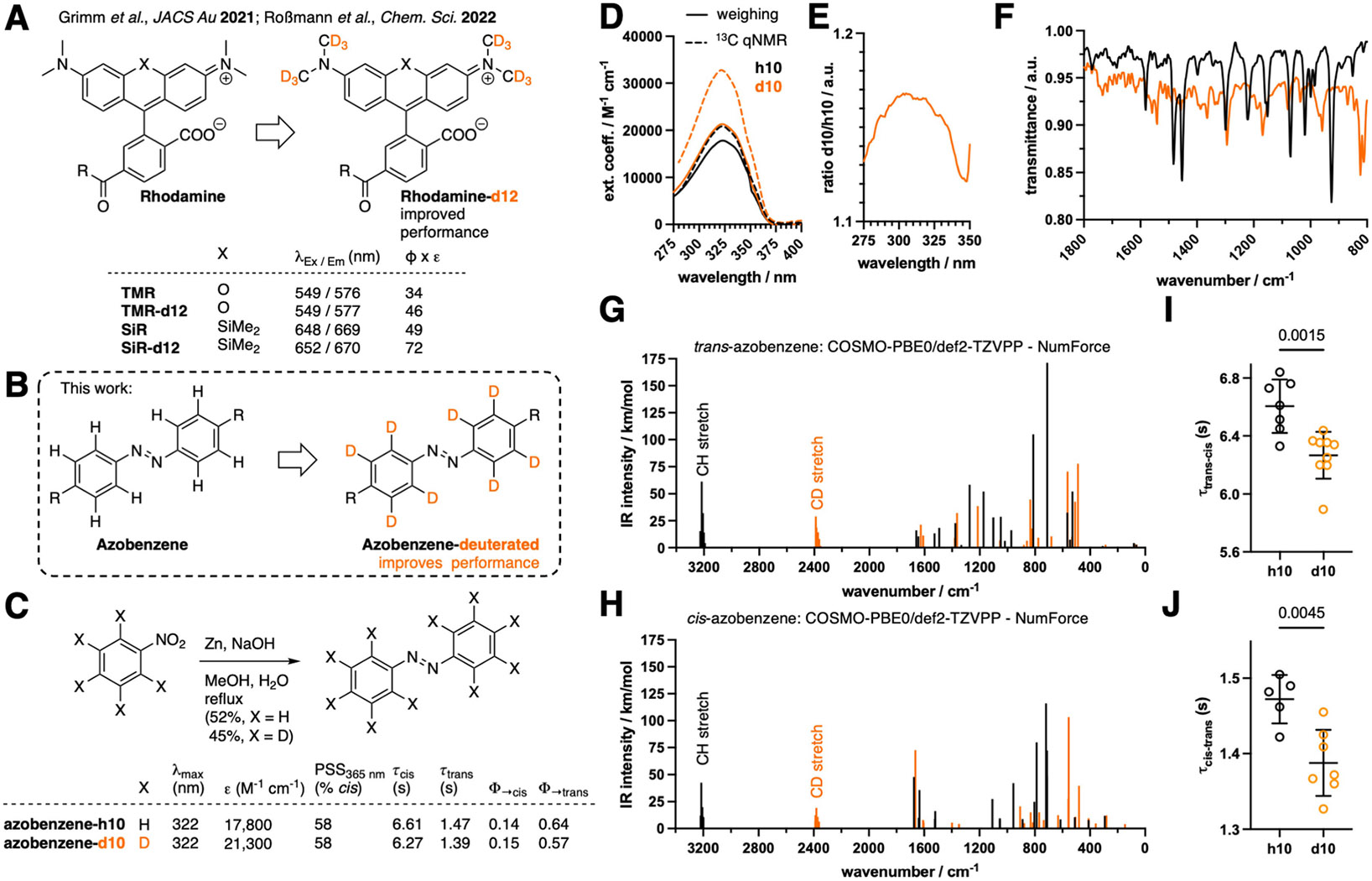
Deuteration strategy to enhance photophysical properties. **A)** Enhanced fluorophore performance was previously obtained by installing deuterated *N*-methyl rhodamines. While excitation/emission spectra show minute changes, brightness (Φ×ε) is drastically enhanced by deuteration. **B)** In this work, we extend this concept by perdeuterating azobenzene chromophores. **C**) Synthesis of azobenzene-d10 by reductive dimerization of nitrobenzene using zinc. Extinction coefficient and switching kinetics are increased by deuteration. **D**) UV/Vis spectra and extinction coefficient determination of azobenzene-h10 and azobenzene-d10 in DMSO by ^13^C qNMR (dashed lines) or by weighing (solid lines). **E**) Ratio of azobenzene-d10 and azobenzene-h10 shows different absorbance characteristics. **F**) IR spectra of *trans*-azobenzene-d10 shows distinct vibrational states in the fingerprint region. **G**) Calculated IR spectra for *trans*-azobenzene-d10 and *trans*-azobenzene-h10 also display different vibrational states. **H**) Calculated IR spectra for *cis*-azobenzene-d10 and *cis*-azobenzene-h10 display different vibrational states. **I, J**) Switching kinetics for *trans*-to-*cis* (I) and *cis*-to-*trans* (J) photoconversion of azobenzene-h10 and azobenzene-d10 in DMSO (100 uM). 2–4 isomerization curves were measured in duplicate and fitted individually. Data represent mean ± SD. P-values from unpaired t-tests are reported in panel I, J.

Despite their utility, all of the aforementioned techniques involve chemical modifications to the underlying compound, thus altering the core structure of the molecule. This makes the process of improving photopharmacological ligands laborious and molecule-specific and raises the possibility that chemical modifications will not be tolerated due to constraints of the target molecule’s binding site. Methods that can be broadly applied to any azobenzene-based system without the need for compound-specific engineering are, thus, needed. One such option is to pursue deuteration, which introduces isotope effects without altering the structure of the chromophore itself. This strategy is motivated by recent studies showing that replacing α-carbon hydrogens of rhodamines or methine protons of cyanines with deuterium can enhance fluorophore performance^[34-36]^ via an increase in molar extinction coefficients and fluorescence quantum yields (Figure 1A). The increase in fluorescence quantum yields is largely due to a reduction of the rate of internal conversion to the ground state. This rate depends on available vibrations, and as the vibrational energy for C─D is smaller than for C─H, a higher vibrational energy level of the electronic ground state is needed to be reached in the relaxation process, reducing its rate. As the rate of non-radiative relaxation is important both for achieving a high photon emission as well as photoisomerization quantum yield, we asked if similar improvements may be obtained with azobenzene photoswitch chromophores (Figure 1B). Deuterated azobenzenes have been described before to investigate drug metabolism,^[37]^ to study ^13^C shifts in NMR spectroscopy,^[38]^ or to obtain “IR clean” switches.^[39]^

## Results and Discussion

We first aimed for the simplest model system, a ‘naked’ azobenzene with either 10 hydrogens (“AB-h10”) or 10 deuteriums (“AB-d10”) (Figure 1B, R=D). Synthesis was straightforwardly achieved from non-deuterated or deuterated nitrobenzene using zinc as a reducing agent in refluxing methanol, and the desired azobenzenes were obtained in 51 % and 45 % yields, respectively (Figure 1C). We characterized the photophysical properties of these molecules and found that the maximal absorbance wavelength in dimethyl sulfoxide (DMSO) remained unchanged at 322 nm (Figure 1C,D). To determine extinction coefficients via Lambert-Beer’s Law, a titration series is usually measured and concentration is plotted against absorbance. Exact concentrations are therefore necessary to be determined if the origin (i.e. zero absorbance at zero concentration) is to be taken into account. Since no hydrogen atoms are present in azobenzene-d10, we performed hydrogen-coupled, quantitative ^13^C NMR to determine concentrations using *N,N*-dimethylformamide (DMF) as an internal standard and found using UV/Vis spectroscopy that the extinction coefficient was increased by >50 % (ε_322 nm_ = 20,800 versus 32,000 M^−1^ cm^−1^) for AB-d10 (Figure 1D). Impressed by this change, and to exclude distortions by nuclear Overhauser effects due to different nuclei, we confirmed this trend by weighing each compound and observed the extinction coefficient to be increased by ~20 % due to deuteration (ε_322 nm_ = 17,800 versus 21,300 M^−1^ cm^−1^) (Figure 1C, D). We note that in both cases, AB-h10 was close to reported literature values of 22,400 M^−1^ cm^−1^ at 319 nm in methanol.^[40]^ Interestingly, by plotting the absorbance of the deuterated azobenzene divided by the absorbance of the non-deuterated azobenzene, we observed a subtle change in spectra around the maximal absorbance peak (Figure 1C, E), indicating different vibrational states for the two molecules. To further examine this, we recorded IR spectra of AB-h10 and AB-d10 and found, as expected, differences in the fingerprint region (Figure 1F). In order to obtain more information on the vibrational states, we performed density functional theory calculation of AB-h10 and AB-d10 at the PBE0^[41,42]^/def2-TZVPP^[43]^ level of theory with the conductor-like screening model (COSMO)^[44]^ to mimic a DMSO solution, finding that the CH/CD stretching bands are shifted by ~800 cm^−1^ for both the *trans*- (Figure 1G, Supplemental Figure 1A and Supplemental Table 1) and *cis*-isomer (Figure 1H, Supplemental Figure 1B and Supplemental Table 2). Deuteration did not affect the photostationary state, which we assessed by integrating absorbance peaks via liquid chromatography-mass spectrometry (LCMS) after irradiating AB-h10 and AB-d10 with 365 nm light, resulting in 58 % *cis*-AB in both cases (Figure 1C). For optical probes in a bioimaging context, the brightness (ε×Φ_F_) is used as a benchmark, which can in a photopharmacological context be translated to sensitivity (molar absorptivity×Φ_switch_). To assess photoisomerization efficiency, we obtained *trans*-to-*cis* switching quantum yields, and found a non-significant trend toward higher quantum yield for AB-d10 (15 %) versus AB-h10 (14 %) in DMSO (Figure 1C, and see Supporting Information). Measuring *cis*-to-*trans* switching quantum yields, we observed a drop for AB-d10 (57 %) versus AB-h10 (64 %) in DMSO (Figure 1C, and see Supporting Information) by excitation at the isosbestic point to be more accurate than extracting values from the *trans*-to-*cis* fits (see Supporting Information for detail). While no changes within error limits of the photoisomerization quantum yield of h10/d10 were found (ref SI), the molar absorptivity of d10 was enhanced, resulting in a total increase of the sensitivity. Interestingly, deuterated nitrobenzyl photocages have been reported with a rate deceleration of more than 8-fold, stemming from a lower reaction quantum yield compared to the non-deuterated counterparts.^[45]^ Remarkably, due to this increase in our case, an acceleration in the *trans*-to-*cis* photoconversion should be gained, which is most relevant to potential photopharmacological applications. Indeed, we observed a clear acceleration in *trans*-to-*cis* (τ = 6.61 vs. 6.27 sec with 3.21 mW/mm^2^ at 365 nm) and *cis*-to-*trans* (τ = 1.47 vs. 1.39 sec with 17.2 mW/mm^2^ at 460 nm) photoswitching kinetics for 100 μM azobenzene-d10 in DMSO (Figure 1I,J). Since photoconversion rates are measured in bulk and kinetic orders are dependent on multiple factors (concentration, extinction, quantum yield, irradiation source etc.), we confirmed the trend of faster switching of AB-d10 at different parameters by using first-order exponential switching: by using a diluted 10 μM solution in DMSO and applying different intensities and different wavelengths (2.14 mW/mm^2^ at 365 nm; 8.29 mW/mm^2^ at 500 nm) (Supplemental Figure 1C).

**Figure 2.**
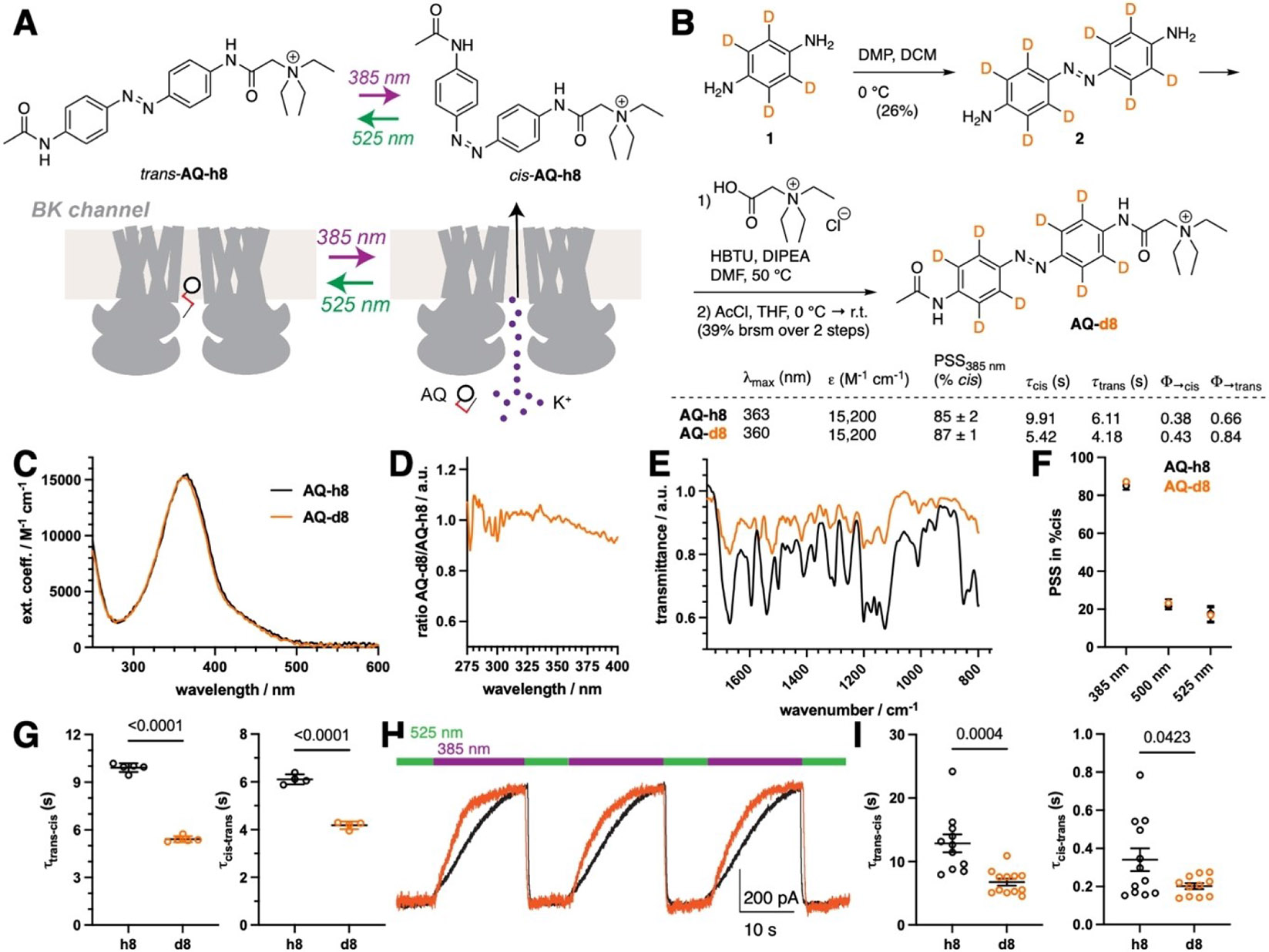
Deuteration enhances performance of a soluble, photoswitchable potassium channel blocker. **A)** *trans*-AQ blocks potassium channels at an intracellular site and unblocking can be achieved optically by applying 385 nm light, with reversibility using 525 nm light. **B**) Synthesis of AQ-d8 and summary of photophysical properties. **C**) UV/Vis spectra and extinction coefficient determination of AQ-h8 and AQ-d8 in water by ^1^H qNMR. **D**) Ratio of AQ-h8 and AQ-d8 shows different absorbance characteristics. **E**) IR spectra of AQ-h8 (black) and AQ-d8 (orange) show different vibrational states. **F**) Photostationary state occupancies under different wavelengths as assessed by ^1^H qNMR. **G**) in vitro Switching kinetics of AQ-h8 and AQ-d8 in water (100 μM). 2–4 isomerization curves were measured in duplicate and fitted individually. Data represent mean ± SD. **H**) BK channel representative trace at +60 mV in response to 385 nm (purple) and 525 nm (green) light in the presence of AQ-h8 (black) or AQ-d8 (orange). **I**) Quantification of channel photo-unblock (left) and photo-block (right) kinetics. 11–12 cells were patched for each AQ variant in three separate biological replicates. Data represent mean ± SEM. P-values from unpaired t-tests are reported in panels G and I.

Encouraged by the above results indicating that deuteration can improve azobenzenes, we pursued a water soluble azobenzene-based compound since organic solvent effects do not recapitulate the cellular environment where most photoswitches are, ultimately, applied. We chose azobenzene quaternary ammonium (AQ) as a scaffold that is bis-amidated and carries a positive charge for excellent water solubility. AQ has been used with various substituents to optically control potassium channels in a plethora of studies on nociception, vision restoration, and neuromodulation (Figure 2A) .^[13,23,46-50]^ We synthesized deuterated AQ-d8 by oxidatively dimerizing phenylene diamine-d4 (**1**) with Dess–Martin periodinane to obtain a perdeuterated 4,4’-bisamine azobenzene **2**, before HBTU-mediated coupling to betaine and subsequent acylation using acetyl chloride was carried out (Figure 2B). We profiled AQ-h8 and AQ-d8 and found similar maximal absorbance at 363 nm and 360 nm (Figure 2C), respectively. We determined extinction coefficients in water to be 15,200 M^−1^ cm^−1^ for both compounds via ^1^H qNMR using DMF as an internal standard (Figure 2C). We had to neglect accurate weighing of the compounds for this assay as the hygroscopic nature of AQs does not permit this, however, we benefited from integrating ^1^H instead of relying on ^13^C as for AB, which is deemed more accurate. We probed the change in UV/Vis absorbance by measuring the ratio of values for AQ-h8 and AQ-d8 and found subtle changes around the maximal absorbance value (Figure 2D). Astonishingly, we found a substantial difference in *trans*-to-*cis* quantum yields,^[51]^ where AQ-d8 (43 %) outperformed AQ-h8 (38 %) and in *cis*-to-*trans* quantum yields where AQ-d8 (84 %) also outperformed AQ-h8 (66 %) (Figure 2C and see Supporting Information). As the measured molar absorptivity of these two compounds is the same, the overall sensitivity was, thus, increased for AQ-d8. IR spectra also showed distinct shifts in vibrational motions (Figure 2E), indicating differences due to the deuterium isotopes. ^1^H qNMR measurements allowed us to determine photostationary states (as described previously)^[23]^ in D_2_O under 385 nm, 500 nm and 525 nm irradiation where similar values were seen for both compounds (Figure 2F). Important in a photopharmacological setting, we observed that switching kinetics were much faster for AQ-d8 than AQ-h8 (*trans*-to-*cis*: τ = 9.91 vs. 5.42 sec; *cis*-to-*trans*: τ = 6.11 vs. 4.18 sec) at 100 μM in water (Figure 2G), which was recapitulated at 10 μM (see Supporting Information). As such, the photo-physically observed increase in the sensitivity of these compounds is directly evident in faster macroscopic switching kinetics. Encouraged by this, we tested the ability of AQ-h8 and AQ-d8 to control the activity of large conductance voltage and calcium-gated (BK) potassium channels via patch-clamp electrophysiology in HEK293 cells. We delivered 1 mM of AQ-h8 or AQ-d8 to the cytosol via the patch pipette and observed robust, reversible photo-block and photo-unblock by illuminating successively with 525 nm and 385 nm light (Figure 2H). While the efficiency of photo-block was similar for AQ-h8 and AQ-d8 (Figure S1A, B), AQ-d8 showed substantially faster macroscopically observed photoswitch kinetics (Figure 2I). Importantly, given that AQ acts as a simple pore blocker, photocurrent kinetics likely serve as a direct readout of *cis/trans* switching kinetics.

**Figure 3.**
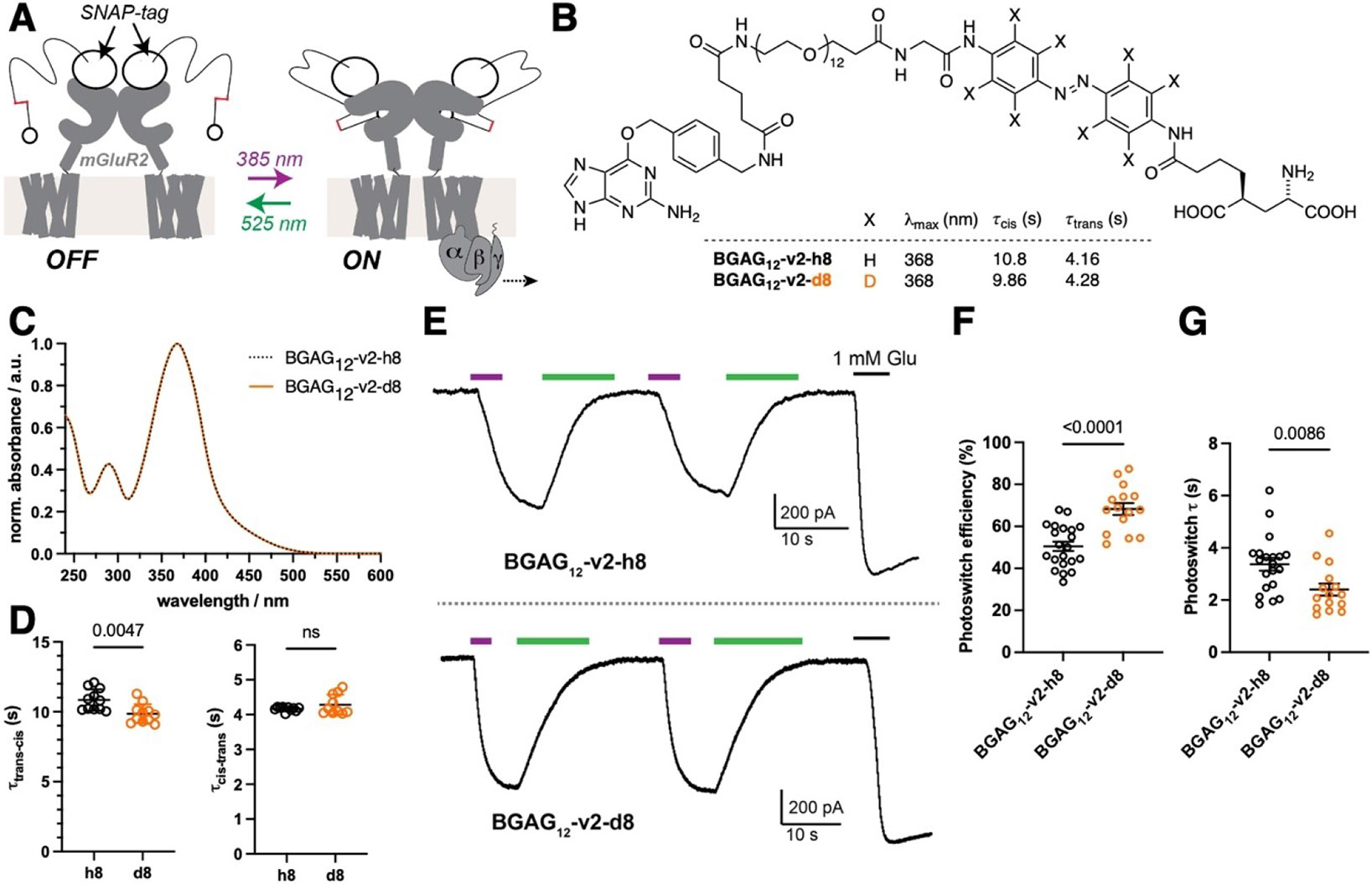
Deuteration enables more efficient, faster optical control of a GPCR via a tethered photoswitch. **A**) Schematic showing optical control of mGluR2-mediated G protein signalling via the PORTL “BGAG”. BGAG is attached to an *N*-terminal fused SNAP-tag and activates mGluR2 upon 385 nm light and can be turned off using 525 nm light. **B**) Structure of BGAG_12_-v2-h8/d8 including in vitro photophysical and kinetic properties. **C**) Normalized UV/Vis spectra BGAGs acquired by LCMS. **D**) Switching kinetics for *trans*-to-*cis* (left) and *cis*-to-*trans* (right) photoconversion of BGAG12-v2-h8 and BGAG12-v2-d8 in water (100 μM). 5–6 isomerization curves were measured in duplicate and fitted individually. Data represent mean ± SD. **E**) GIRK current recordings of SNAP-mGluR2 photoswitching reveals reversibility and repeatability of switching, and increased performance of BGAG_12_-v2-d8. **F**) Quantified photoswitch efficiency for BGAG_12_-v2-h8/d8. **G**) Kinetics of SNAP-mGluR2 photoswitching. 15–22 cells were patched for each BGAG variant in three or four biological replicates. Data represent mean ± SEM. P-values from unpaired t-tests reported in panels F and G.

Photopharmacology can be merged with the power of genetic engineering by tethering photoswitchable ligands to a self-labelling tag (e.g. SNAP) on a protein of interest.^[32]^ This approach yields excellent target selectivity due to the bioorthogonal nature of labelling and rapid G protein-coupled receptor (GPCR) photoactivation kinetics due to the lack of ligand diffusion. We previously pioneered this approach by conjugating the SNAP-tagged metabotropic glutamate receptor 2 (SNAP-mGluR2), a neuromodulatory GPCR, with a “photoswitchable orthogonal remotely-tethered ligand“ (PORTL) which enables rapid, reversible optical control of mGluR2 activity ex vivo and in vivo (Figure 3A).^[23,33,52]^ The PORTL ligand consists of a benzylguanine-azobenzene-glutamate (“BGAG”) photoswitch, with BGAG_12_-v2-h8 serving as a testbed for our deuteration strategy. Employing a previously described synthetic route (Figure 3B; Supporting Information), we obtained deuterated BGAG_12_-v2-d8, which showed the same maximal absorbance wavelength of BGAG_12_-v2-h8 (Figure 3B; Figure S2A). BGAG_12_-v2-d8 labelled SNAP-mGluR2 transfected HEK293 cells with the same efficiency as its non-deuterated counterpart (Figure S2B, C). Testing for photophysical and kinetic properties, we found no change in absorbance between non- and deuterated BGAG_12_-v2 (Figure 3C), which was to be assumed since AQ bears the same azobenzene substitution pattern, i.e. a 4,4’-*N*-amide linker. Similar to AQ-d8, we found enhanced *trans*-to-*cis* kinetics of BGAG_12_-v2-d8 over BGAG_12_-v2-h8 at 100 μM in water (Figure 3D), but *cis*-to-*trans* remained unaffected. This trend was confirmed using 10 μM of each BGAG (Supporting Information). Moving into a biological system by using patch-clamp electrophysiology with G protein-coupled inward-rectifying potassium (GIRK) channels as a reporter, we observed robust and reversible responses by applying 385 nm (ON) and 525 nm (OFF) light (Figure 3E). When comparing photocurrents to the response to a saturating concentration of glutamate (1 mM), a clear increase in photoswitching efficiency was observed from ~50 % to ~68 % for BGAG_12_-v2-d8 (Figure 3F). In addition, we observed a faster ON response when 385 nm light was applied (Figure 3G). It should be noted that OFF kinetics, which do not change between BGAG_12_-v2-d8 and BGAG_12_-v2-h8, do not recapitulate photoswitch kinetics in this system but are limited by biological signal termination processes. Nevertheless, the PORTL system allows for a clean readout since photoswitch concentration is determined by receptor expression level.

## Conclusions

In summary, we have translated the deuteration strategy from fluorophores to azobenzene photoswitches, where we find substantially improved properties in terms of sensitivity (molar extinction coefficient, photoisomerization, quantum yield), which results in faster macroscopic photoswitching kinetics. More than 80 years after the report of *cis*-azobenzene,^[53]^ the complex photophysics of azobenzene switches remain under debate on the theoretical and spectroscopic level,^[54-56]^ and future work is needed to fully decipher the underlying mechanism. While this may be achieved with ultrafast spectroscopy and advanced molecular modelling, in this study we focused on the utility of this strategy for photopharmacological applications. We demonstrate the ability of deuteration to enhance azobenzene photoswitching on two distinct systems, a soluble photochromic ligand (AQ) and a tethered PORTL (BGAG), suggesting that this strategy can be widely applicable to the many azobenzene scaffolds and ligands which have been reported. We note that while our paper was in review, a publication from Li and colleagues described a deuterated azocholine with improved properties for the optical control of α7 nicotinic receptors, highlighting the importance of our concept.^[57]^ Interestingly, while all three compounds reported here showed clearly improved photoswitch kinetics, there was variability in the extent of the effects on light absorbance and photoswitching efficiency, motivating future analyses of the vast chemical space for deuteration (or semideuteration) on the aromatic units of an azobenzene and/or their substituents (e.g. *N*-methyl amine deuteration) to further optimize this strategy.

## Supplementary Material

SupplementaryMaterial

## Data Availability

The data that support the findings of this study are available from the corresponding author upon reasonable request.
