# Supplementary material for: Deuteration as a General Strategy to Enhance Azobenzene-Based Photopharmacology
Source: Angew Chem Int Ed Engl. Author manuscript; Available in PMC 2025 May 5. (PMC12051094; doi:10.1002/anie.202408300)
Supplement: SupplementaryMaterial [file NIHMS2074597-supplement-SupplementaryMaterial.pdf]

Angewandte Chemie
Eine Zeitschrift der Gesellschaft Deutscher Chemiker

Supporting Information

Deuteration as a General Strategy to Enhance Azobenzene-Based Photopharmacology

K. Roßmann, A. J. Gonzalez-Hernandez, R. Bhuyan, C. Schattenberg, H. Sun, K. Börjesson, J. Levitz, J. Broichhagen**

Supplemental Information for:

Deuteration as a General Strategy to Enhance Azobenzene-based Photopharmacology

Kilian Roßmann[1,#], Alberto J. Gonzalez-Hernandez[2,#], Rahul Bhuyan[3], Caspar Schattenberg[1], Han Sun[1], Karl Börjesson[3], Joshua Levitz[2,*], Johannes Broichhagen[1,*]

[1] Leibniz-Forschungsinstitut für Molekulare Pharmakologie (FMP), 13125 Berlin, Germany.

[2] Department of Biochemistry, Weill Cornell Medicine, New York, NY 10065, USA

[3] Department of Chemistry and Molecular Biology, University of Gothenburg, 413 90 Gothenburg, Sweden

[#] Equal contribution

* Correspondence should be addressed to:
jtl2003@med.cornell.edu and broichhagen@fmp-berlin.de

Table of Contents

1. General 4
2. Synthesis 5
2.1. (*E*)-1,2-Diphenyldiazene (AB-h10) 5
2.2. (*E*)-1,2-Bis(phenyl-d$_5$)diazene (AB-d10) 5
2.3. (*E*)-4,4'-(Diazene-1,2-diyl)bis(benzen-2,3,5,6-d$_4$-amine) (2) 6
2.4. (*E*)-2-((4-((4-Aminophenyl-2,3,5,6-d$_4$)diazenyl)phenyl-2,3,5,6-d$_4$)amino)-*N,N,N*-triethyl-2-oxoethan-1-aminium 6
2.5. (*E*)-2-((4-((4-Acetamidophenyl-2,3,5,6-d$_4$)diazenyl)phenyl-2,3,5,6-d$_4$)amino)-*N,N,N*-triethyl-2-oxoethan-1-aminium (AQ-d8) 7
2.6. (Dimethyl (2*S*,4*S*)-2-(4-((4-((*E*)-(4-aminophenyl-2,3,5,6-d4)diazenyl)phenyl-2,3,5,6-d4)amino)-4-oxobutyl)-4-((*tert*-butoxycarbonyl)amino)pentanedioate (4) 8
2.7. Dimethyl (2*S*,4*S*)-2-(4-((4-((*E*)-(4-(2-aminoacetamido)phenyl-2,3,5,6-d4)diazenyl)phenyl-2,3,5,6-d4)amino)-4-oxobutyl)-4-((*tert*-butoxycarbonyl)amino)pentanedioate (5) 9
2.8. Dimethyl (2*S*,4*S*)-2-(4-((4-((*E*)-(4-(1-amino-39-oxo-3,6,9,12,15,18,21,24,27,30,33,36-dodecaoxa-40-azadotetracontan-42-amido)phenyl-2,3,5,6-d4)diazenyl)phenyl-2,3,5,6-d4)amino)-4-oxobutyl)-4-((*tert*-butoxycarbonyl)amino)pentanedioate (6) 10
2.9. (*E*)-2-((4-((4-Acetamidophenyl-2,3,5,6-d4)diazenyl)phenyl-2,3,5,6-d4)amino)-*N,N,N*-triethyl-2-oxoethan-1-aminium Dimethyl (2*S*,4*S*)-2-(4-((4-((*E*)-(4-(1-(4-(((2-amino-9*H*-purin-6-yl)oxy)methyl)phenyl)-3,7,47-trioxo-11,14,17,20,23,26,29,32,35,38,41,44-dodecaoxa-2,8,48-triazapentacontan-50-amido)phenyl-2,3,5,6-d4)diazenyl)phenyl-2,3,5,6-d4)amino)-4-oxobutyl)-4-((*tert*-butoxycarbonyl)amino)pentanedioate (7) 11
2.10 .... (2*S*,4*S*)-2-Amino-4-(4-((4-((*E*)-(4-(1-(4-(((2-amino-9*H*-purin-6-yl)oxy)methyl)phenyl)-3,7,47-trioxo-11,14,17,20,23,26,29,32,35,38,41,44-dodecaoxa-2,8,48-triazapentacontan-50-amido)phenyl-2,3,5,6-d4)diazenyl)phenyl-2,3,5,6-d4)amino)-4-oxobutyl)pentanedioic acid (BGAG$_{12}$-v2-d8) 12
2.11.... (*E*)-2-((4-((4-Acetamidophenyl-2,3,5,6-d4)diazenyl)phenyl-2,3,5,6-d4)amino)-*N,N,N*-triethyl-2-oxoethan-1-aminium Dimethyl (2*S*,4*S*)-2-(4-((4-((*E*)-(4-(1-(4-(((2-amino-9*H*-purin-6-yl)oxy)methyl)phenyl)-3,7,47-trioxo-11,14,17,20,23,26,29,32,35,38,41,44-dodecaoxa-2,8,48-triazapentacontan-50-amido)phenyl)diazenyl)phenyl)amino)-4-oxobutyl)-4-((*tert*-butoxycarbonyl)amino)pentanedioate (8) 13
2.12 .... (2*S*,4*S*)-2-Amino-4-(4-((4-((*E*)-(4-(1-(4-(((2-amino-9*H*-purin-6-yl)oxy)methyl)phenyl)-3,7,47-trioxo-11,14,17,20,23,26,29,32,35,38,41,44-dodecaoxa-2,8,48-triazapentacontan-50-amido)phenyl)diazenyl)phenyl)amino)-4-oxobutyl)pentanedioic acid (BGAG$_{12}$-v2-h8) 14
3. NMR spectroscopy 15
3.1. (*E*)-1,2-Bis(phenyl-d5)diazene 15
3.2. (*E*)-1,2-Diphenyldiazene 16
3.3. (*E*)-4,4'-(Diazene-1,2-diyl)bis(benzen-2,3,5,6-d$_4$-amine) 17
3.4. (*E*)-2-((4-((4-Aminophenyl-2,3,5,6-d$_4$)diazenyl)phenyl-2,3,5,6-d$_4$)amino)-*N,N,N*-triethyl-2-oxoethan-1-aminium 18
3.5. (*E*)-2-((4-((4-Acetamidophenyl-2,3,5,6-d$_4$)diazenyl)phenyl-2,3,5,6-d$_4$)amino)-*N,N,N*-triethyl-2-oxoethan-1-aminium (AQ-d8) 19
3.6. (2*S*,4*S*)-2-Amino-4-(4-((4-((*E*)-(4-(1-(4-(((2-amino-9*H*-purin-6-yl)oxy)methyl)phenyl)-3,7,47-trioxo-11,14,17,20,23,26,29,32,35,38,41,44-

dodecaoxa-2,8,48-triazapentacontan-50-amido)phenyl-2,3,5,6-d4)diazenyl)phenyl-2,3,5,6-d4)amino)-4-oxobutyl)pentanedioic acid (BGAG$_{12}$-v2-d8)....................................20
3.7. (2*S*,4*S*)-2-Amino-4-(4-((4-((*E*)-(4-(1-(4-(((2-amino-9*H*-purin-6-yl)oxy)methyl)phenyl)-3,7,47-trioxo-11,14,17,20,23,26,29,32,35,38,41,44-dodecaoxa-2,8,48-triazapentacontan-50-amido)phenyl)diazenyl)phenyl)amino)-4-oxobutyl)pentanedioic acid (BGAG$_{12}$-v2-h8)....................................21
3.8. Overlay of ^{1}H NMR spectra of BGAG$_{12}$-v2-h8 (blue) and BGAG$_{12}$-v2-d8 (red).......21
4. DFT Calculations....................................22
5. Photochemical actinometry....................................25
6. Photoisomerization quantum yield measurements....................................26
7. Supplementary Figures....................................32
8. Supplementary Schemes....................................34
9. Cell culture, molecular biology and patch clamp electrophysiology....................................35
10. References....................................35

1. General

All chemical reagents and anhydrous solvents for synthesis were purchased from commercial suppliers (Sigma-Aldrich, Fluka, Acros, Fluorochem, TCI) and were used without further purification.

NMR spectra were recorded in deuterated solvents on a Bruker AVANCE III 600 equipped with a CryoProbe or on a Bruker AVANCE II 750 and calibrated to residual solvent peaks ($^1H/^{13}C$ in ppm): D_2O (4.70), DMSO-d_6 (2.50/39.52), MeOD-d_4 (3.31/49.00). Multiplicities are abbreviated as follows: s = singlet, d = doublet, t = triplet, q = quartet, p = pentet, h = heptet, br = broad, m = multiplet. Coupling constants *J* are reported in Hz. Spectra are reported based on appearance, not on theoretical multiplicities derived from structural information. Light for switching was delivered by a CoolLED pE-4000 (intensities used: $I_{385\ nm}$ = 7.21 mW/mm^2; $I_{500\ nm}$ = 4.14 mW/mm^2; $I_{525\ nm}$ = 2.50 mW/mm^2). Full spectra were acquired either in the dark or under appropriate illumination, and photostationary states were recorded at one wavelength under annotated illumination.

UPLC-UV/Vis for purity assessment was performed on an Agilent 1260 Infinity II LC System equipped with Agilent SB- C18 column (1.8 µm, 2.1 × 50 mm). Buffer A: 0.1% FA in H2O Buffer B: 0.1% FA acetonitrile. The typical gradient was from 10% B for 0.5 min → gradient to 95% B over 5 min → 95% B for 0.5 min → gradient to 99% B over 1 min with 0.8 mL/min flow. Retention times (tR) are given in minutes (min). Chromatograms were imported into Graphpad Prism10 and plotted.

High resolution ESI-MS spectra were recorded on a Waters H-class instrument equipped with a quaternary solvent manager, a Waters sample manager-FTN, a Waters PDA detector and a Waters column manager with an Acquity UPLC protein BEH C18 column (1.7 µm, 2.1 mm x 50 mm). Samples were eluted with a flow rate of 0.3 mL/min. The following gradient was used: A: 0.01 % FA in H_2O; B: 0.01 % FA in MeCN. 5 % B: 0-1 min; 5 to 95 % B: 1-7min; 95 % B: 7 to 8.5 min. Mass analysis was conducted with a Waters XEVO G2-XS QTof analyzer.

Reactions and chromatography fractions were monitored by thin layer chromatography (TLC) on Merck silica gel 60 F254 glass plates. The spots were visualized either under UV light at 254 nm and/or 366 nm or with appropriate staining method (iodine, *para*-anisaldehyde, $KMnO_4$) followed by heating.

UV/Vis spectroscopy was performed on either: i) a JASCO V-550 UV/Vis/NIR spectrophotometer or ii) a Thermo ScientificTM NanoDropTM OneC microvolume-UV/Vis-spectrophotometer using a Hellma quartz glass cuvette (10 mm pathlength). Light for switching was delivered by a CoolLED pE-4000 (intensities used: $I_{365\ nm}$ = 2.14 mW/mm^2 or 3.21 mW/mm^2; $I_{385\ nm}$ = 0.72 mW/mm^2; $I_{460\ nm}$ = 17.2 mW/mm^2; $I_{500\ nm}$ = 8.29 mW/mm^2). Full spectra were acquired either in the dark or under appropriate illumination, and kinetic traces were recorded at 320 nm (for AB) or 365 nm (for AQ and BGAG$_{12}$-v2) under annotated illumination. Results were exported and plotted in GraphPad Prism 10 and kinetic traces were fitted monoexponentially GraphPad Prism 10 and plotted in GraphPad Prism 10.

IR spectroscopy was performed on a Bruker Tensor 27 FT-IR and evaluated with OPUS 8.5.

2. Synthesis

2.1. (*E*)-1,2-Diphenyldiazene (AB-h10)

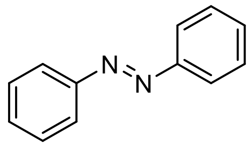

A 50 mL flask was charged with nitrobenzene (96 mg, 781 µmol, 1.0 equiv.) in 10 mL methanol, and a solution of sodium hydroxide (101.5 mg, 1.56 mmol, 2.0 equiv.) dissolved in 3 mL H_2O was added, before zinc dust (125.0 mg, 3.12 mmol, 4.0 equiv.) was added and the reaction was stirred for 12 h under reflux. The mixture was filtered while hot, and concentrated *in vacuo*. The residue was again dissolved in 2 mL hot methanol, and dried onto isolute. The mixture was subjected to column chromatography, with a mixture of EtOAc/cyclohexane (1:99). The product was concentrated *in vacuo* to obtain 37.0 mg (203 µmol) of the desired compound as an orange powder in 52% yield.

^{1}H NMR (600 MHz, MeOD-d_4): δ [ppm] = 7.93 (d, J = 7.4 Hz, 4H), 7.57 (t, J = 7.4 Hz, 4H), 7.53 (t, J = 7.2 Hz, 2H).
^{13}C NMR (150 MHz, MeOD-d_4): δ [ppm] = 152.6, 130.9, 128.9, 122.4.
HRMS (ESI): calc. for $C_{12}H_{10}N_2$ $[M+H]^+$: 183.0917, found: 183.0956.
IR (wavenumber / cm^{-1}): 3063.7, 2925.3, 1957.4, 1899.0, 1808.2, 1771.3, 1685.4, 1582.2, 1539.7, 1483.1, 1453.3, 1398.0, 1299.4, 1221.7, 1151.2, 1071.2, 1019.7, 999.7, 985.4, 926.0, 851.2

2.2. (*E*)-1,2-Bis(phenyl-d_5)diazene (AB-d10)

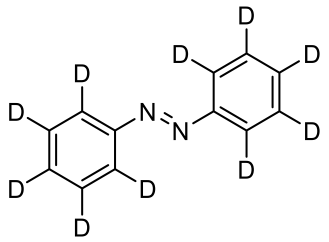

A 50 mL flask was charged with 1-nitrobenzene-2,3,4,5,6-d_5 (100 mg, 781 µmol, 1.0 equiv.) in 10 mL methanol, and a solution of sodium hydroxide (101.5 mg, 1.56 mmol, 2.0 equiv.) dissolved in 3 mL H_2O was added, before zinc dust (125.0 mg, 3.12 mmol, 4.0 equiv.) was added and the reaction was stirred for 12 h under reflux. The mixture is filtered while hot, and concentrated *in vacuo*. The residue was again dissolved in 2 mL hot methanol, and put on isolute. The mixture was subjected to column chromatography, with a mixture of EtOAc/cyclohexane (1:99). The product was concentrated *in vacuo* to obtain 34.0 mg (177 µmol) of the desired compound as an orange powder in 45% yield.

^{1}H NMR (600 MHz, MeOD-d_4): δ [ppm] = no peaks observed.
^{13}C NMR (150 MHz, MeOD-d_4): δ [ppm] = 152.5, 130.5 (t, J = 24.0 Hz), 128.5 (t, J = 24.6 Hz), 122.0 (t, J = 24.6 Hz).
HRMS (ESI): calc. for $C_{12}D_{10}N_2$ [M+H]+: 193.1544, found: 193.1531
IR (wavenumber / cm^{-1}): 3853.9, 3838.6, 3735.6, 3670.2, 3648.3, 2926.3, 2853.3, 2270.5, 1733.6, 1616.6, 1541.6, 1457.5, 1364.3, 1294.1, 1168.7, 1080.0, 1035.7, 958.8, 868.8, 823.4, 811.3

2.3. (*E*)-4,4'-(Diazene-1,2-diyl)bis(benzen-2,3,5,6-d_4-amine) (2)

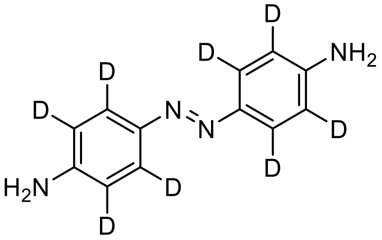

A round bottom flask was charged with benzene-d_4-1,4-diamine (**1**, 100 mg, 893 µmol, 1.0 equiv.) and dissolved in 50 mL CH_2Cl_2. To the vigorously stirred solution was added Dess-Martin-periodinane (832 mg, 1.96 mmol, 2.2 equiv.) dissolved in 50 mL CH_2Cl_2 over 90 min, and the mixture was stirred for an additional 30 min. The mixture was subjected directly to column chromatography to first elute CH_2Cl_2, before the residue was purified with a mixture of EtOAc/cyclohexane (50:50). The solution was concentrated *in vacuo* and subjected to an additional purification by RP-HPLC to obtain 28.0 mg (127 µmol) of the desired compound as an orange powder in 28% yield after lyophilization.

^{1}H NMR (600 MHz, DMSO-d_6): δ [ppm] = no peaks observed.
^{2}H NMR (92 MHz, DMSO-d_6): δ [ppm] = 7.54 (s, 4D), 6.64 (s, 4D).
^{13}C NMR (150 MHz, DMSO-d_6): δ [ppm] = 147.7, 144.5, 123.5 (t, J = 24.1 Hz), 115.1 (t, J = 23.6 Hz).
HRMS (ESI): calc. for $C_{12}H_4D_8N_4$ $[M+H]^+$: 221.1637, found: 221.1647.

2.4. (*E*)-2-((4-((4-Aminophenyl-2,3,5,6-d_4)diazenyl)phenyl-2,3,5,6-d_4)amino)-*N,N,N*-triethyl-2-oxoethan-1-aminium

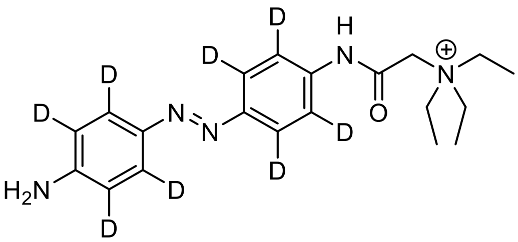

A 50 mL round bottom flask was charged with *E*)-4,4'-(diazene-1,2-diyl)bis(benzen-2,3,5,6-d_4-amine (**2**, 14 mg, 63.6 µmol, 1.0 equiv.), *N*-(carboxymethyl)-*N,N*-diethylethanaminium chloride (Banghart et al., 2009) (30.5 mg, 191 µmol, 3.0 equiv.), dissolved in 1 mL DMSO, before 86 µL DIPEA was added. To the vigorously stirred solution was added HBTU (79.5 mg 210 µmol, 3.3 equiv.) in one portion and the reaction mixture was stirred at 50 °C o.n. before it was quenched by addition of 200 µL HOAc and 200 µL H_2O and subjected to RP-HPLC purification to obtain 5.9 mg (16.3 µmol) of the desired product in 26% yield, while 43% of the starting material were recovered.

^{1}H NMR (600 MHz, DMSO-d_6): δ [ppm] = 10.9 (s, 1H), 4.20 (s, 2H), 3.55 (q, J = 7.2 Hz, 6H), 1.28 (t, J = 7.2 Hz, 9H).
^{13}C NMR (150 MHz, DMSO-d_6): δ [ppm] = 162.4, 152.8, 149.3, 143.2, 138.9, 125.1–124.7 (m), 123.0–122.4 (m), 120.5–120.0 (m), 114.0–113.4 (m), 56.8, 54.5, 7.88.
HRMS (ESI): calc. for $C_{20}H_{20}D_8N_5O^+$ $[M]^+$: 362.2791, found: 362.2758.

2.5. (*E*)-2-((4-((4-Acetamidophenyl-2,3,5,6-d$_4$)diazenyl)phenyl-2,3,5,6-d$_4$)amino)-*N,N,N*-triethyl-2-oxoethan-1-aminium (AQ-d8)

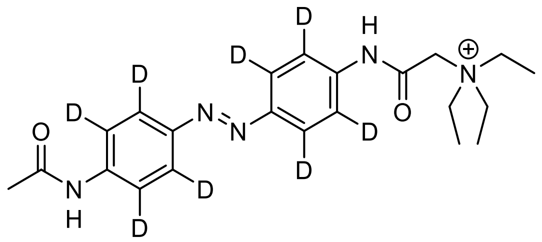

A flame-dried 50 mL Schlenk flask under an argon atmosphere was charged with (*E*)-2-((4-((4-aminophenyl-2,3,5,6-d$_4$)diazenyl)phenyl-2,3,5,6-d$_4$)amino)-*N,N,N*-triethyl-2-oxoethan-1-aminium (1.5 mg, 4.14 µmol, 1.0 equiv.) and dissolved in 1 mL dry THF, before 1.31 µL DIPEA (8.28 µmol, 2.0 equiv.) was added. The reaction mixture was vigorously stirred and cooled to 0 °C in an ice-bath, before AcCl (0.5 µL, 6.21 µmol, 1.5 equiv.) was added. Upon addition, the ice bath was removed and the reaction mixture was allowed to stir for 30 min while warming to r.t., before it was quenched by addition of glacial HOAc (10 µL). The reaction mixture was concentrated *in vacuo* and subjected to RP-HPLC to obtain 0.9 mg (2.23 µmol) of the desired compound (TFA salt) as a highly hygroscopic, orange powder in 54% yield.

^{1}H NMR (600 MHz, D_2O): δ [ppm] = 4.09 (s, 2H), 3.57 (q, J = 7.2 Hz, 6H), 1.32 (t, J = 7.2 Hz, 9H).
^{13}C NMR (150 MHz, D_2O): δ [ppm] = 172.9, 162.8, 149.2, 148.6, 140.2, 138.7, 56.5, 54.7, 23.1, 7.0, 4 carbons could not be observed.
HRMS (ESI): calc. for $C_{22}H_{22}D_8N_5O_2^+$ [M]$^+$: 404.2897, found: 404.2898.
IR (wavenumber / cm^{-1}): 3838.4, 3750.4, 3648.9, 2365.3, 1670.1, 1567.9, 1521.5, 1456.9, 1417.9, 1372.8, 1318.0, 1245.3, 1200.1, 1127.4, 1007.3, 833.4

IR (wavenumber / cm^{-1}) for AQ-h8 (ref[1]): 3936.1, 3862.8, 3838.4, 3802.6, 3751.5, 3734.1, 3670.9, 3332.7, 2364.6, 1684.5, 1541.1, 1115.9, 1078.9, 1012.1, 842.5

2.6. (Dimethyl (2*S*,4*S*)-2-(4-((4-((*E*)-(4-aminophenyl-2,3,5,6-d4)diazenyl)phenyl-2,3,5,6-d4)amino)-4-oxobutyl)-4-((*tert*-butoxycarbonyl)amino)pentanedioate (4)

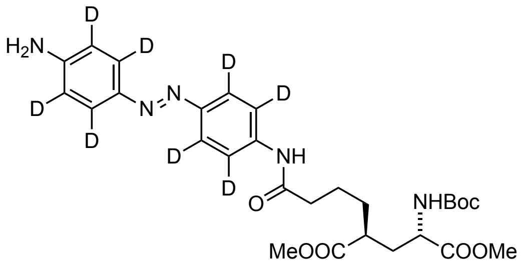

A round bottom flask was charged with (5*S*,7*S*)-7-((*tert*-butoxycarbonyl)amino)-8-methoxy-5-(methoxycarbonyl)-8-oxooctanoic acid (3, 32 mg, 90.9 µmol, 1.0 equiv.), 2 (20 mg, 90.9 µmol, 1.0 equiv.) and dissolved in DMSO (2 mL) and DIPEA (47 mg, 364 µmol, 63 µL, 4.0 equiv.) before HBTU (35 mg, 90.9 µmol, 1.0 equiv.) was added. The reaction mixture was allowed to incubate at r.t. for 4 hours before it was quenched with HOAc (63 µL) and water (100 µL) and subjected to RP-HPLC purification. The product containing fractions were pooled and lyophilized to obtain 21 mg (37.3 µmol) of the desired product in 41% yield as a red powder.

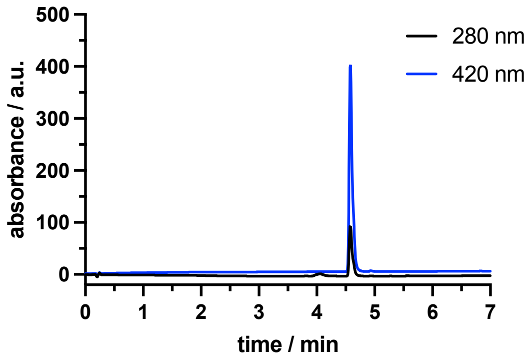

LRMS (ESI): calc. for $C_{28}H_{30}D_8N_5O_7^+$ $[M+H]^+$: 564.3, found: 564.3.

2.7. Dimethyl (2*S*,4*S*)-2-(4-((4-((*E*)-(4-(2-aminoacetamido)phenyl-2,3,5,6-d4)diazenyl)phenyl-2,3,5,6-d4)amino)-4-oxobutyl)-4-((*tert*-butoxycarbonyl)amino)pentanedioate (5)

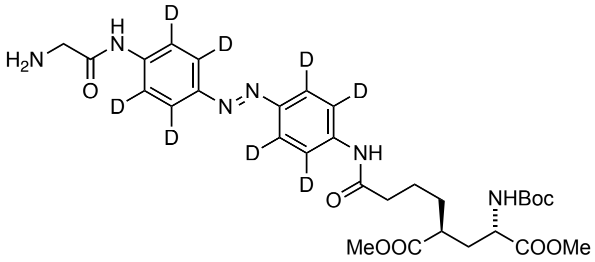

A round bottom flask was charged with (**4**, 21 mg, 37 µmol, 1.0 equiv.), dissolved in dry THF (7 mL) and DIPEA (18 mg, 142 µmol, 25 µL, 4.0 equiv.) and was cooled to 0 °C before FmocGlyCl (22.4 mg, 71 µmol, 1.9 equiv.) was added. The reaction mixture was allowed to incubate at r.t. for 1 hour before DBU in MeCN (150 µL in 5 mL) was added. After 3 hours, the reaction was quenched with HOAc (200 µL) and water (1 mL) and subjected to RP-HPLC purification. The product containing fractions were pooled and lyophilized to obtain 10 µmol of the desired product in 27% yield as an orange powder.

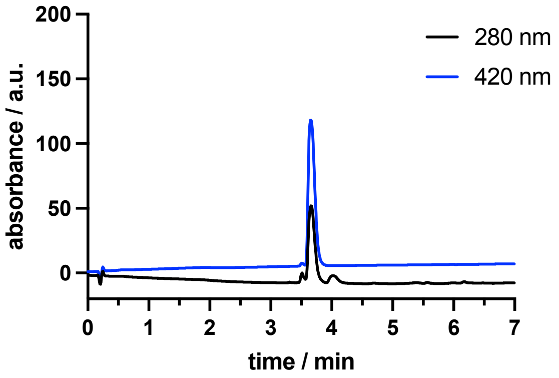

LCMS: *cis*- and *trans*-isomer.
LRMS (ESI): calc. for $C_{30}H_{33}D_8N_6O_8^+$ $[M+H]^+$: 621.3, found: 621.4.

2.8. Dimethyl (2*S*,4*S*)-2-(4-((4-((*E*)-(4-(1-amino-39-oxo-3,6,9,12,15,18,21,24,27,30,33,36-dodecaoxa-40-azadotetracontan-42-amido)phenyl-2,3,5,6-d4)diazenyl)phenyl-2,3,5,6-d4)amino)-4-oxobutyl)-4-((*tert*-butoxycarbonyl)amino)pentanedioate (6)

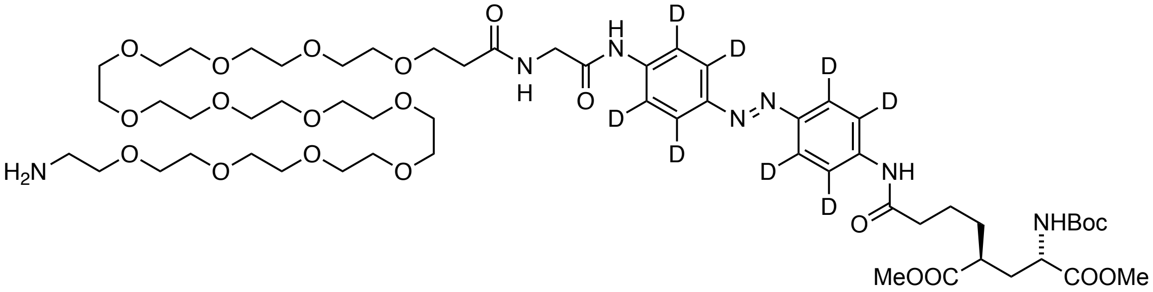

A 1.5 mL Eppendorf vial was charged with 5 (10 µmol, 1.0 equiv.), Fmoc-PEG$_{12}$-COOH (8.4 mg, 10 µmol, 1.0 equiv.) and dissolved in DMF (1 mL) and DIPEA (6.7 mg, 53 µmol, 9.3 µL, 5.3 equiv.) before HBTU (3.8 mg, 10 µmol, 1.0 equiv.) was added. The reaction mixture was allowed to incubate at r.t. for 5 hours before the addition of DBU (50 µL) and let stand for another hour. The reaction was quenched with HOAc (100 µL) and water (200 µL) and subjected to RP-HPLC purification. The product containing fractions were pooled and lyophilized to obtain 8.4 µmol of the desired product in 84% yield as an orange powder.

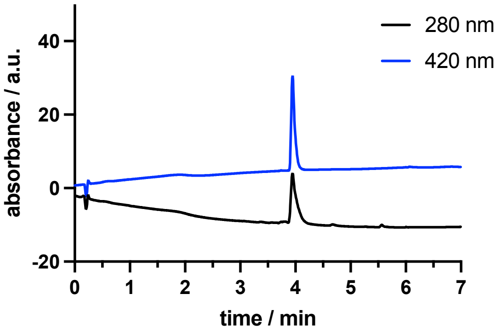

LRMS (ESI): calc. for $C_{57}H_{86}D_8N_7NaO_{21}^{+}$ $[M+H+Na]^{2+}$: 621.8, found: 622.0.

2.9. (*E*)-2-((4-((4-Acetamidophenyl-2,3,5,6-d4)diazenyl)phenyl-2,3,5,6-d4)amino)-*N*,*N*,*N*-triethyl-2-oxoethan-1-aminium Dimethyl (2*S*,4*S*)-2-(4-((4-((*E*)-(4-(1-(4-(((2-amino-9*H*-purin-6-yl)oxy)methyl)phenyl)-3,7,47-trioxo-11,14,17,20,23,26,29,32,35,38,41,44-dodecaoxa-2,8,48-triazapentacontan-50-amido)phenyl-2,3,5,6-d4)diazenyl)phenyl-2,3,5,6-d4)amino)-4-oxobutyl)-4-((*tert*-butoxycarbonyl)amino)pentanedioate (7)

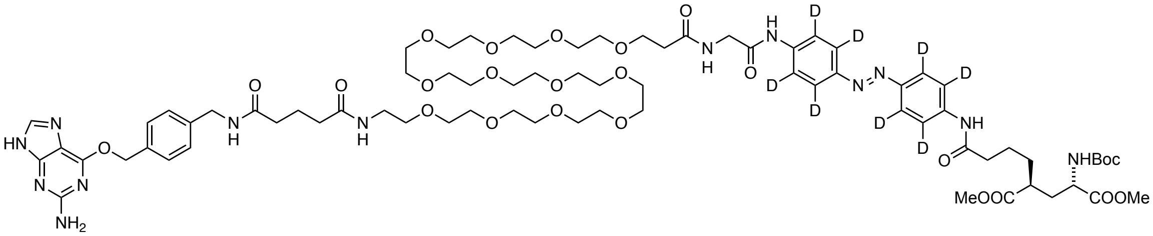

A 1.5 mL Eppendorf vial was charged with 6 (8.4 µmol, 1.0 equiv.), BG-COOH (4.5 mg, 12.6 µmol, 1.5 equiv.) and dissolved in DMF (1 mL) and DIPEA (8.7 mg, 67 µmol, 9.5 µL, 8.0 equiv.) before HBTU (4.8 mg, 12.6 µmol, 1.5 equiv.) was added. The reaction mixture was allowed to incubate at r.t. for 4 hours before it was quenched with HOAc (100 µL) and water (200 µL) and subjected to RP-HPLC purification. The product containing fractions were pooled and lyophilized to obtain 6.0 µmol of the desired product in 71% yield as an orange powder.

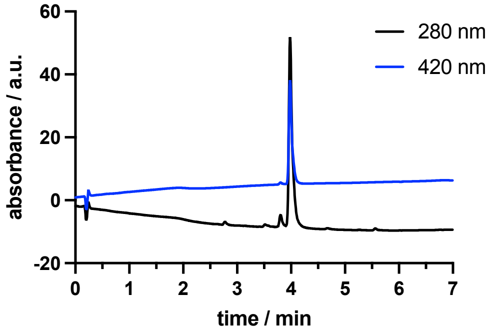

LCMS: *cis*- and *trans*-isomer.
LRMS (ESI): calc. for $C_{75}H_{104}D_8N_{13}O_{24}^{+}$ [M+H]$^{+}$: 1586.8, found: 1586.6.

2.10. (2*S*,4*S*)-2-Amino-4-(4-((4-((*E*)-(4-(1-(4-(((2-amino-9*H*-purin-6-yl)oxy)methyl)phenyl)-3,7,47-trioxo-11,14,17,20,23,26,29,32,35,38,41,44-dodecaoxa-2,8,48-triazapentacontan-50-amido)phenyl-2,3,5,6-d4)diazenyl)phenyl-2,3,5,6-d4)amino)-4-oxobutyl)pentanedioic acid (BGAG$_{12}$-v2-d8)

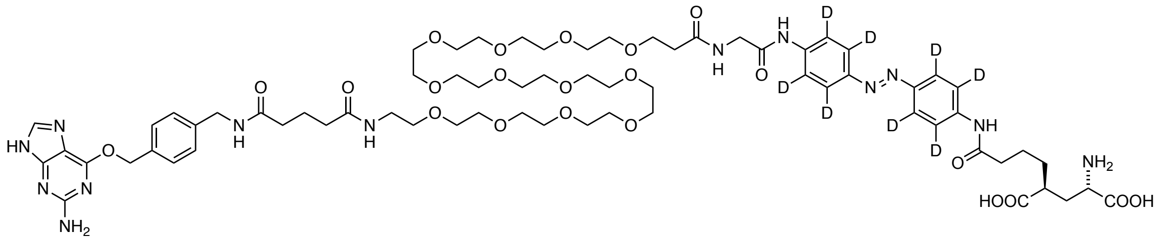

A 15 mL Falcon tube was charged with **7** (6.0 µmol, 1.0 equiv.) and MeOH (250 µL), THF (500 µL) and 1 M LiOH (250 µL) were added sequentially. The reaction mixture was allowed to incubate at r.t. for 2 hours, before volatiles were removed first by a gentle stream of nitrogen and the by lyophilization. The mixture was then cooled to 0 °C and ice-cold TFA (1 mL) was added and the reaction mixture turned deep purple and was allowed to incubate for 1 hour. Removal of the volatiles with a gentle stream of nitrogen by maintaining cooling to 0 °C was performed, before the residue was taken up in 1 mL of MeCN/H_2O/HOAc (25/25/1) and subjected to RP-HPLC purification. The product containing fractions were pooled and lyophilized to obtain 4.7 µmol of the desired product in 78% yield as an orange powder.

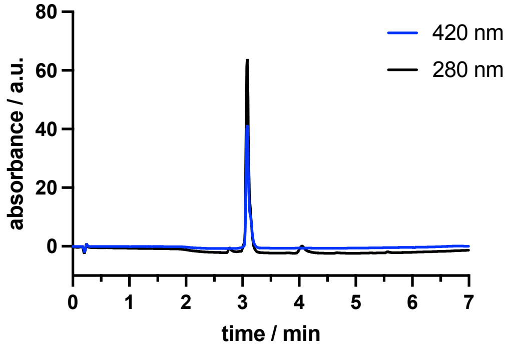

^{1}H NMR (600 MHz, D_2O): δ [ppm] = 7.93 (s, 1H), 7.41 (d, J = 8.0 Hz, 2H), 7.28 (d, J = 8.1 Hz, 2H), 5.35 (s, 2H), 4.30 (s, 2H), 4.07 (s, 2H), 3.80 (t, J = 5.8 Hz, 2H), 3.67 (t, J = 7.1 Hz, 1H), 3.66-3.64 (m, 2H), 3.62-3.59 (m, 2H), 3.58-3.48 (m, 40H), 3.46 (t, J = 5.3 Hz, 2H), 3.24 (t, J = 5.2 Hz, 2H), 2.71-3.65 (m, 1H), 2.62-2.61 (t, J = 5.8 Hz, 2H), 2.42 (t, J = 7.2 Hz 2H), 2.20 (t, J = 7.5 Hz, 2H), 2.14 (t, J = 7.4 Hz, 2H), 2.11-2.03 (m, 1H), 2.01-1.92 (m, 1H), 1.77 (p, J = 7.5 Hz, 2H), 1.73-1.60 (m, 4H).
LRMS (ESI): calc. for $C_{68}H_{92}D_8N_{13}O_{22}^+$ [M+H]$^+$: 1458.8, found: 1459.6.

2.11. (*E*)-2-((4-((4-Acetamidophenyl-2,3,5,6-d4)diazenyl)phenyl-2,3,5,6-d4)amino)-*N*,*N*,*N*-triethyl-2-oxoethan-1-aminium Dimethyl (2*S*,4*S*)-2-(4-((4-((*E*)-(4-(1-(4-(((2-amino-9*H*-purin-6-yl)oxy)methyl)phenyl)-3,7,47-trioxo-11,14,17,20,23,26,29,32,35,38,41,44-dodecaoxa-2,8,48-triazapentacontan-50-amido)phenyl)diazenyl)phenyl)amino)-4-oxobutyl)-4-((*tert*-butoxycarbonyl)amino)pentanedioate (8)

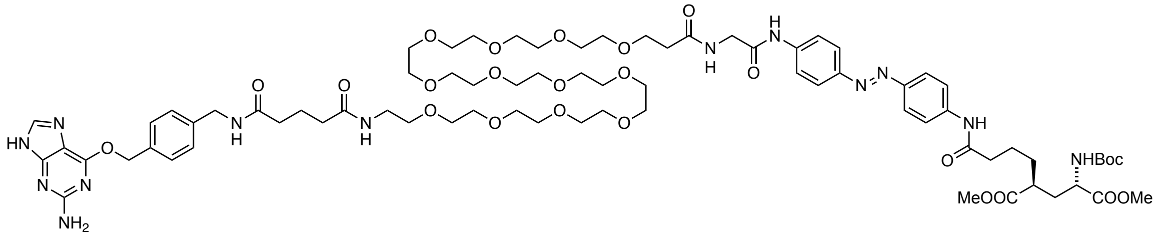

A 1.5 mL Eppendorf vial was charged with dimethyl (2*S*,4*S*)-2-(4-((4-((*E*)-(4-(1-amino-39-oxo-3,6,9,12,15,18,21,24,27,30,33,36-dodecaoxa-40-azadotetracontan-42-amido)phenyl)diazenyl)-phenyl)amino)-4-oxobutyl)-4-((*tert*-butoxycarbonyl)amino)pentanedioate (ref[2]) (22 µmol, 1.0 equiv.), BG-COOH (11.7 mg, 33 µmol, 1.5 equiv.) and dissolved in DMF (2 mL) and DIPEA (23 mg, 176 µmol, 31 µL, 8.0 equiv.) before HBTU (12.5 mg, 33 µmol, 1.5 equiv.) was added. The reaction mixture was allowed to incubate at r.t. for 4 hours before it was quenched with HOAc (100 µL) and water (300 µL) and subjected to RP-HPLC purification. The product containing fractions were pooled and lyophilized to obtain 12.2 µmol of the desired product in 55% yield as an orange powder.

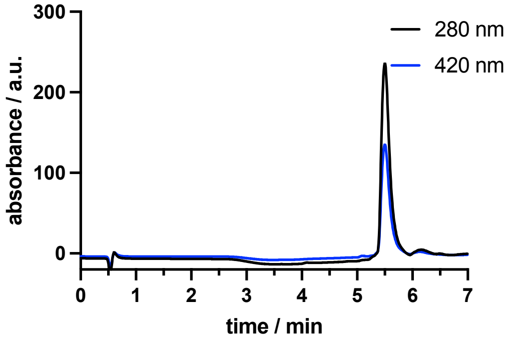

LRMS (ESI): calc. for $C_{75}H_{112}N_{13}O_{24}^{+}$ $[M+H]^{+}$: 1578.8, found: 1578.6.

2.12. (2*S*,4*S*)-2-Amino-4-(4-((4-((*E*)-(4-(1-(4-(((2-amino-9*H*-purin-6-yl)oxy)methyl)phenyl)-3,7,47-trioxo-11,14,17,20,23,26,29,32,35,38,41,44-dodecaoxa-2,8,48-triazapentacontan-50-amido)phenyl)diazenyl)phenyl)amino)-4-oxobutyl)pentanedioic acid (BGAG$_{12}$-v2-h8)

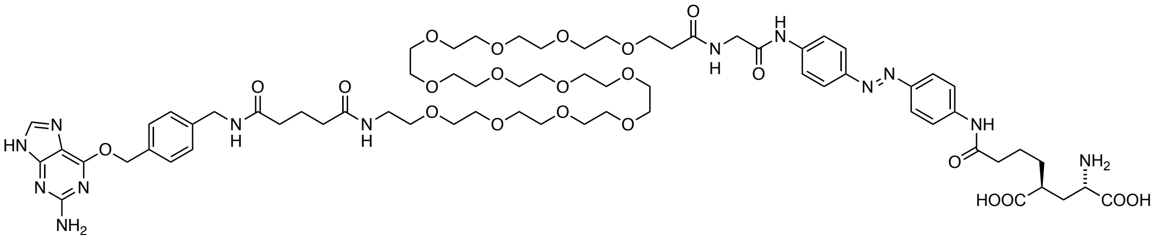

A 15 mL Falcon tube was charged with **8** (12.2 µmol, 1.0 equiv.) and MeOH (250 µL), THF (500 µL) and 1 M LiOH (250 µL) were added sequentially. The reaction mixture was allowed to incubate at r.t. for 2 hours, before volatiles were removed first by a gentle stream of nitrogen and the by lyophilization. The mixture was then cooled to 0 °C and ice-cold TFA (1 mL) was added and the reaction mixture turned deep purple and was allowed to incubate for 1 hour. Removal of the volatiles with a gentle stream of nitrogen by maintaining cooling to 0 °C was performed, before the residue was taken up in 1 mL of MeCN/H_2O/HOAc (25/25/1) and subjected to RP-HPLC purification. The product containing fractions were pooled and lyophilized to obtain 4.1 µmol of the desired product in 34% yield as an orange powder.

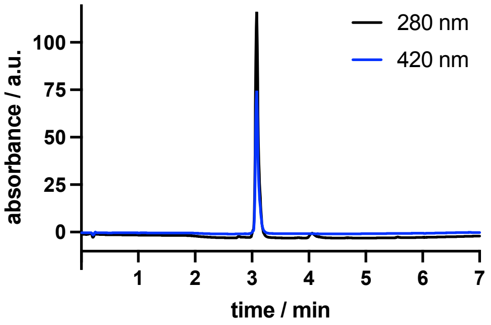

^{1}H NMR (600 MHz, D_2O): δ [ppm] = 7.94 (s, 1H), 7.53-7.45 (m, 8H), 7.40 (d, J = 8.1 Hz, 2H), 7.28 (d, J = 8.0 Hz, 2H), 5.34 (s, 2H), 4.30 (s, 2H), 4.06 (s, 2H), 3.80 (t, J = 5.8 Hz, 2H), 3.68 (t, J = 7.1 Hz, 1H), 3.66-3.64 (m, 2H), 3.61-3.60 (m, 2H), 3.57-3.49 (m, 40H), 3.46 (t, J = 5.3 Hz, 2H), 3.24 (t, J = 5.3 Hz, 2H), 2.71-3.65 (m, 1H), 2.62-2.61 (t, J = 5.8 Hz, 2H), 2.45-2.39 (m, 2H), 2.19 (t, J = 7.5 Hz, 2H), 2.14 (t, J = 7.4 Hz, 2H), 2.11-2.03 (m, 1H), 2.01-1.92 (m, 1H), 1.77 (p, J = 7.5 Hz, 2H), 1.73-1.60 (m, 4H).
LRMS (ESI): calc. for $C_{68}H_{100}N_{13}O_{22}^+$ [M+H]$^+$: 1450.7, found: 1450.0.

3. **NMR spectroscopy**

3.1. (*E*)-1,2-Bis(phenyl-d5)diazene

^{1}H-NMR

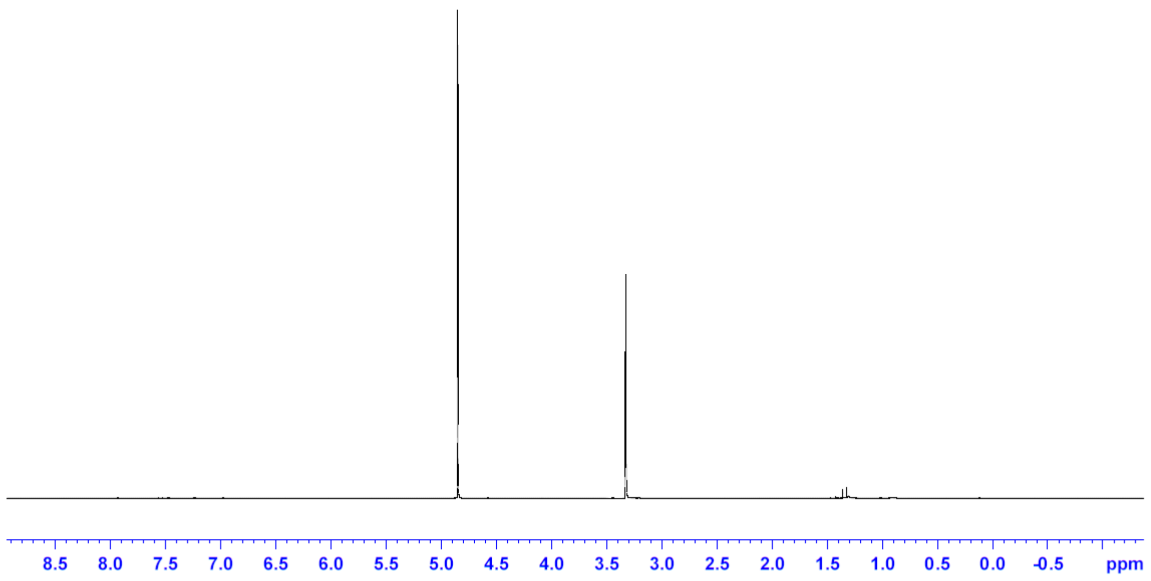

^{13}C-NMR

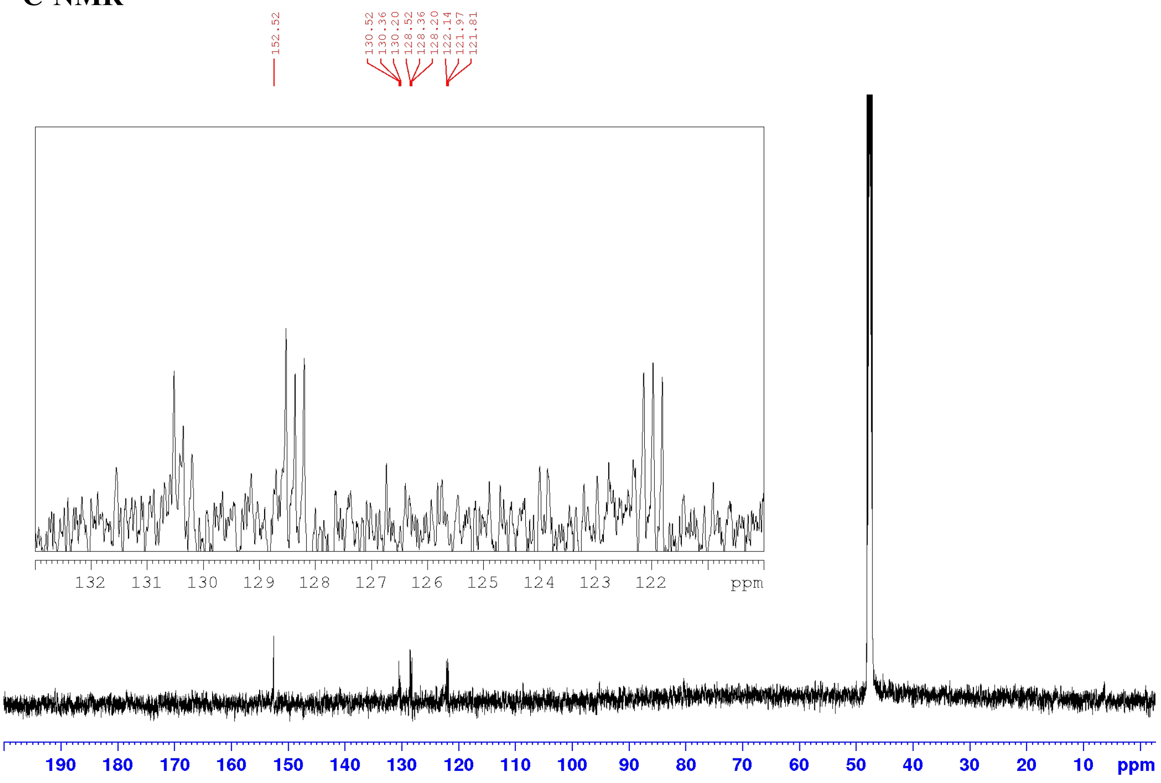

3.2. (*E*)-1,2-Diphenyldiazene

^{1}H-NMR

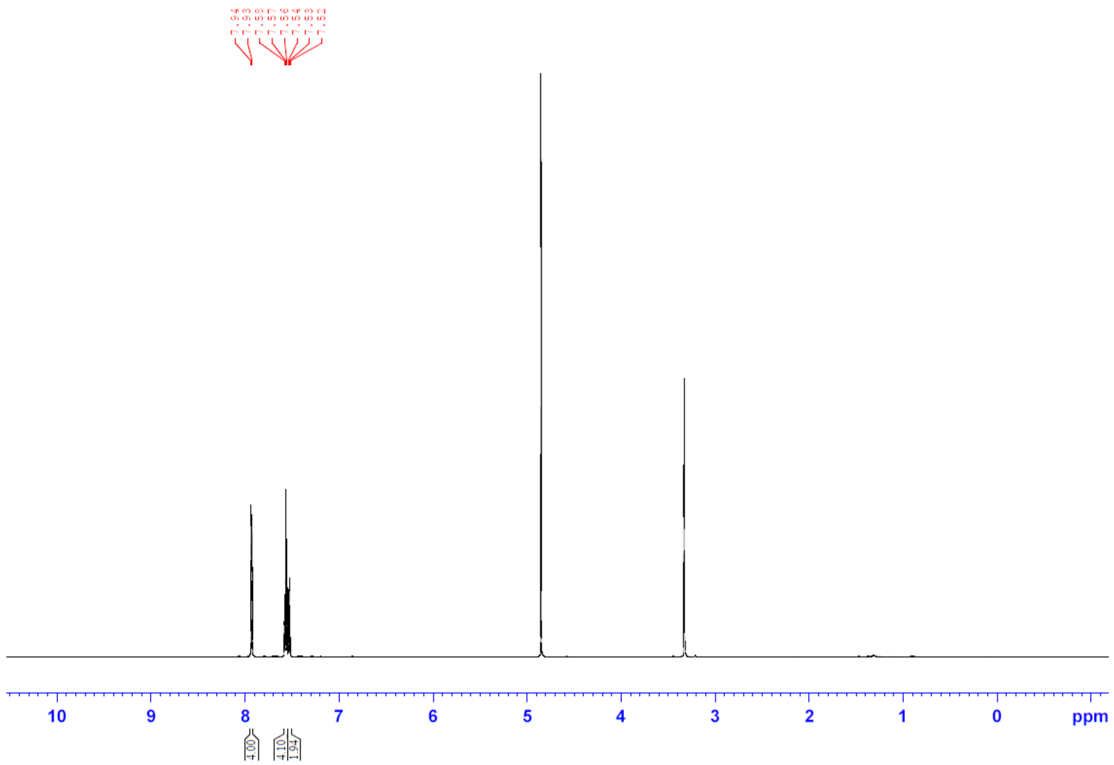

^{13}C-NMR

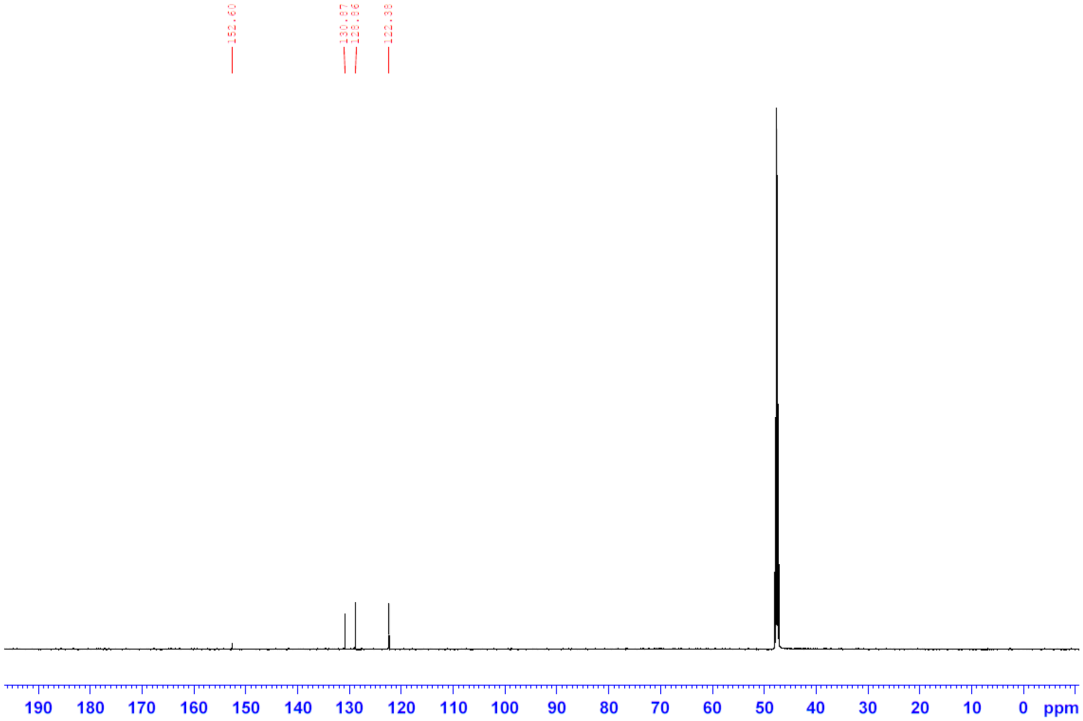

3.3. (*E*)-4,4'-(Diazene-1,2-diyl)bis(benzen-2,3,5,6-d$_4$-amine)

^{2}H-NMR

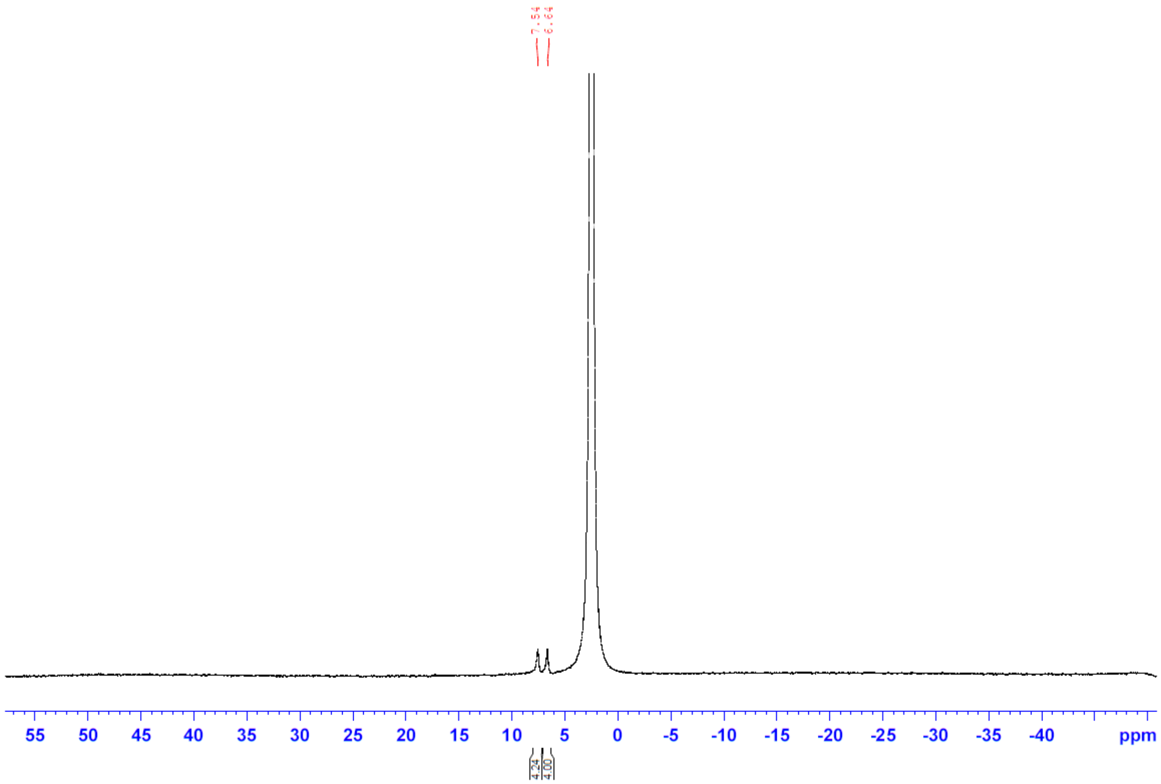

^{13}C-NMR

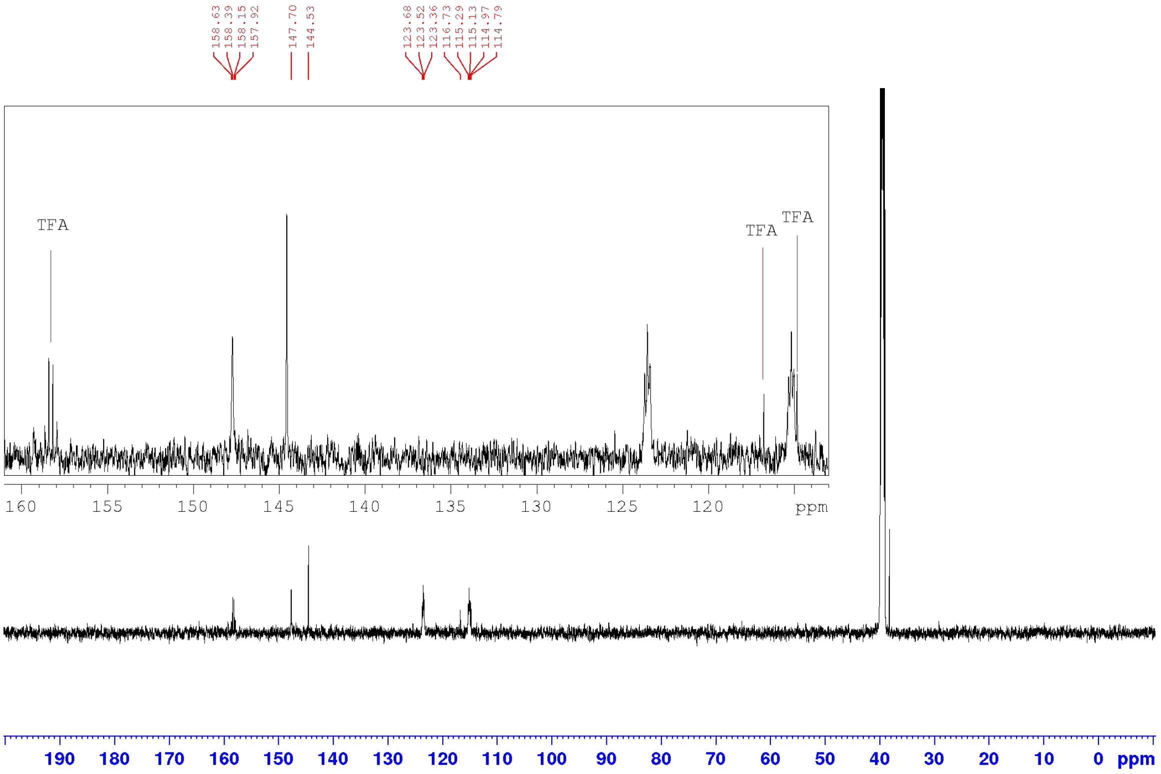

3.4. (*E*)-2-((4-((4-Aminophenyl-2,3,5,6-d4)diazenyl)phenyl-2,3,5,6-d4)amino)-*N,N,N*-triethyl-2-oxoethan-1-aminium

^{1}H-NMR

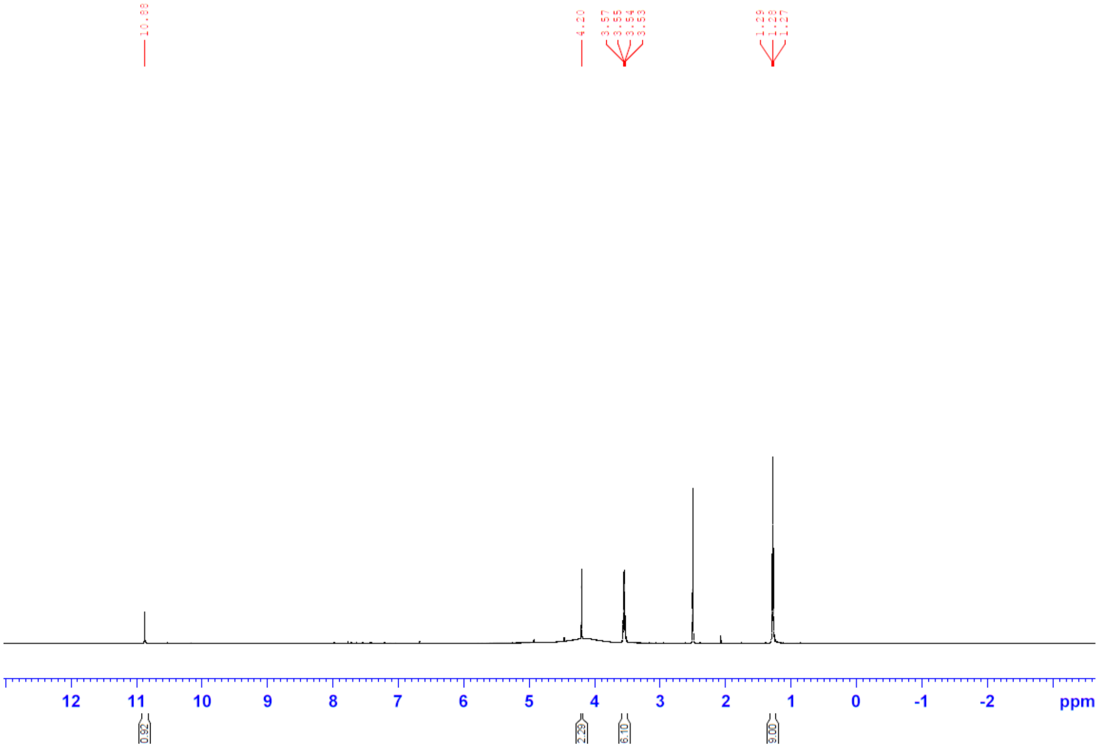

^{13}C-NMR

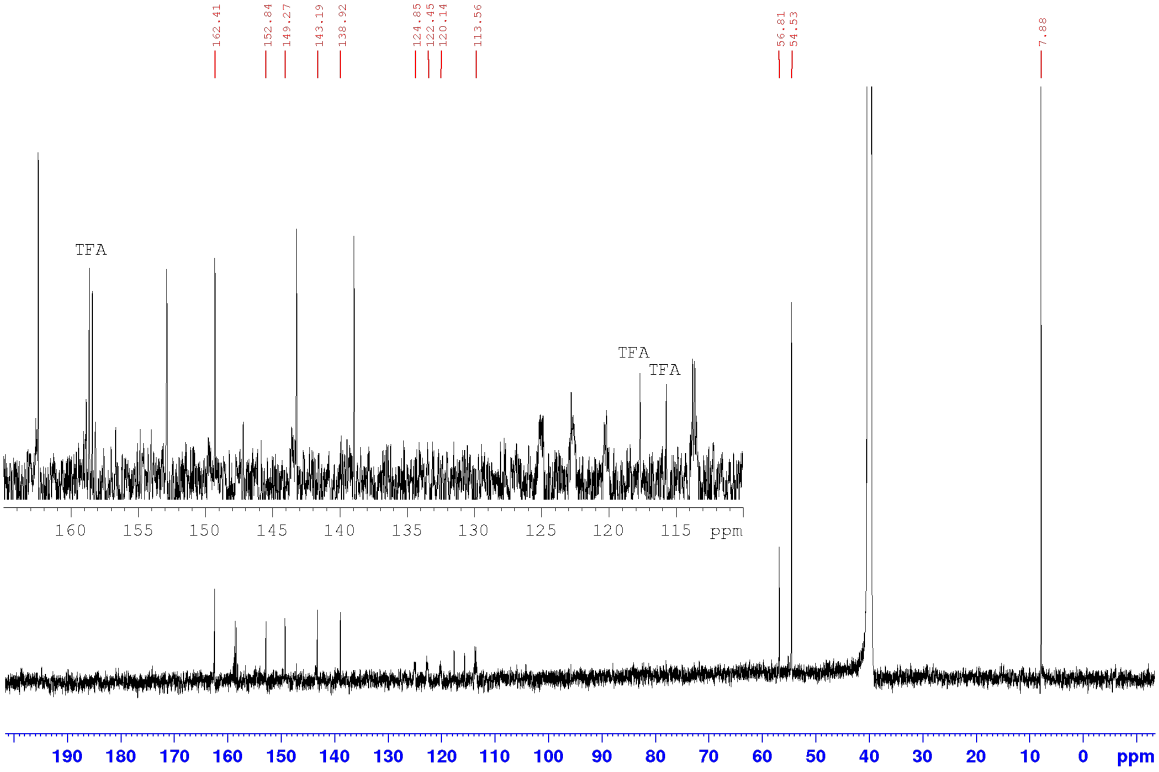

3.5. (*E*)-2-((4-((4-Acetamidophenyl-2,3,5,6-d$_4$)diazenyl)phenyl-2,3,5,6-d$_4$)amino)-*N*,*N*,*N*-triethyl-2-oxoethan-1-aminium (AQ-d8)

^{1}H-NMR

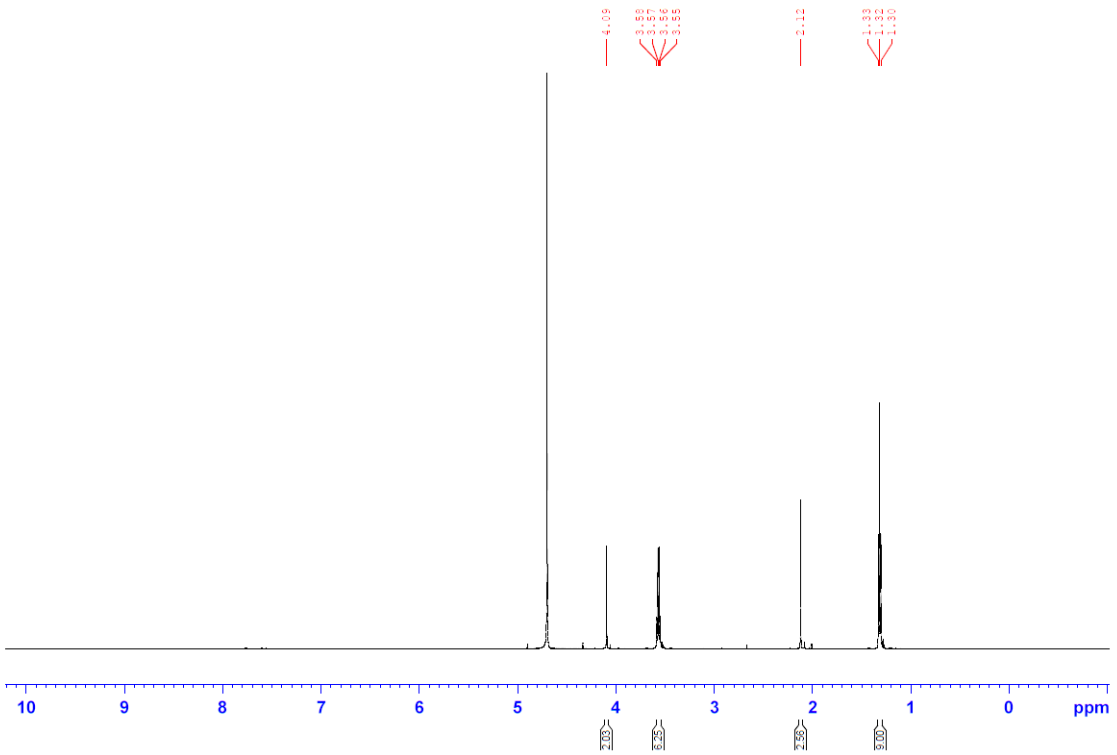

^{13}C-NMR

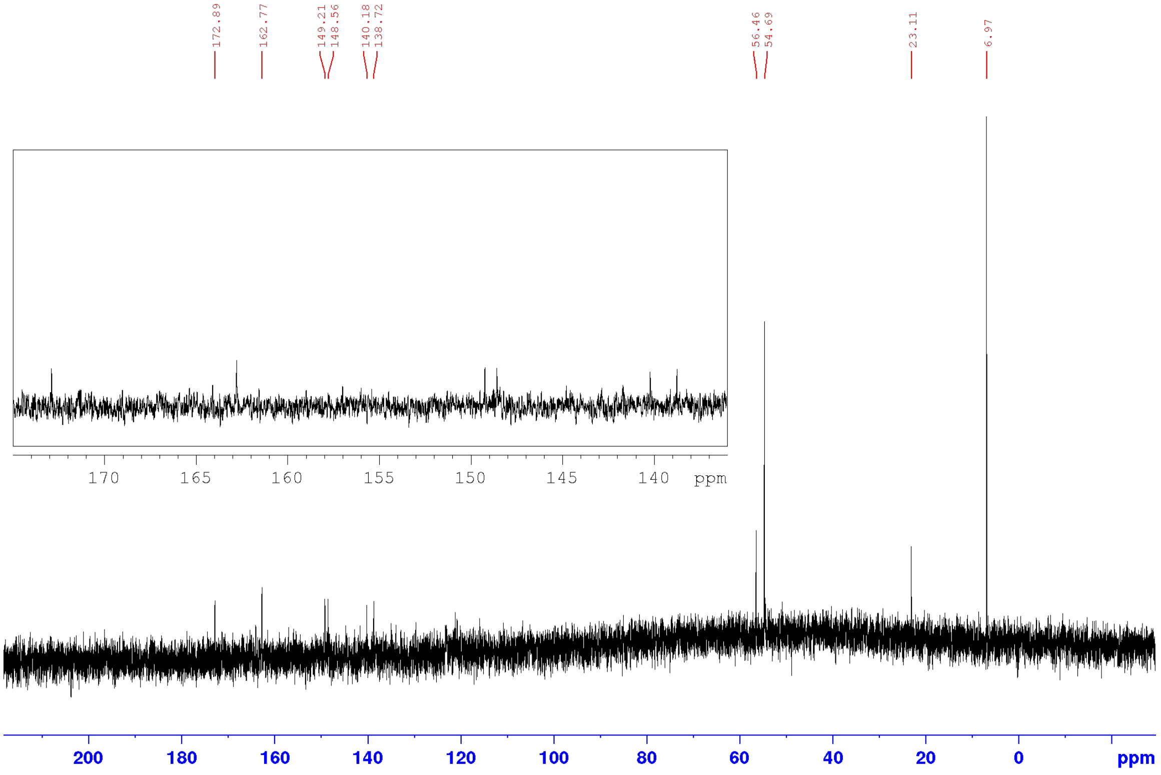

3.6. (2*S*,4*S*)-2-Amino-4-(4-((4-((*E*)-(4-(1-(4-(((2-amino-9*H*-purin-6-yl)oxy)methyl)phenyl)-3,7,47-trioxo-11,14,17,20,23,26,29,32,35,38,41,44-dodecaoxa-2,8,48-triazapentacontan-50-amido)phenyl-2,3,5,6-d4)diazenyl)phenyl-2,3,5,6-d4)amino)-4-oxobutyl)pentanedioic acid (BGAG$_{12}$-v2-d8)

^{1}H-NMR

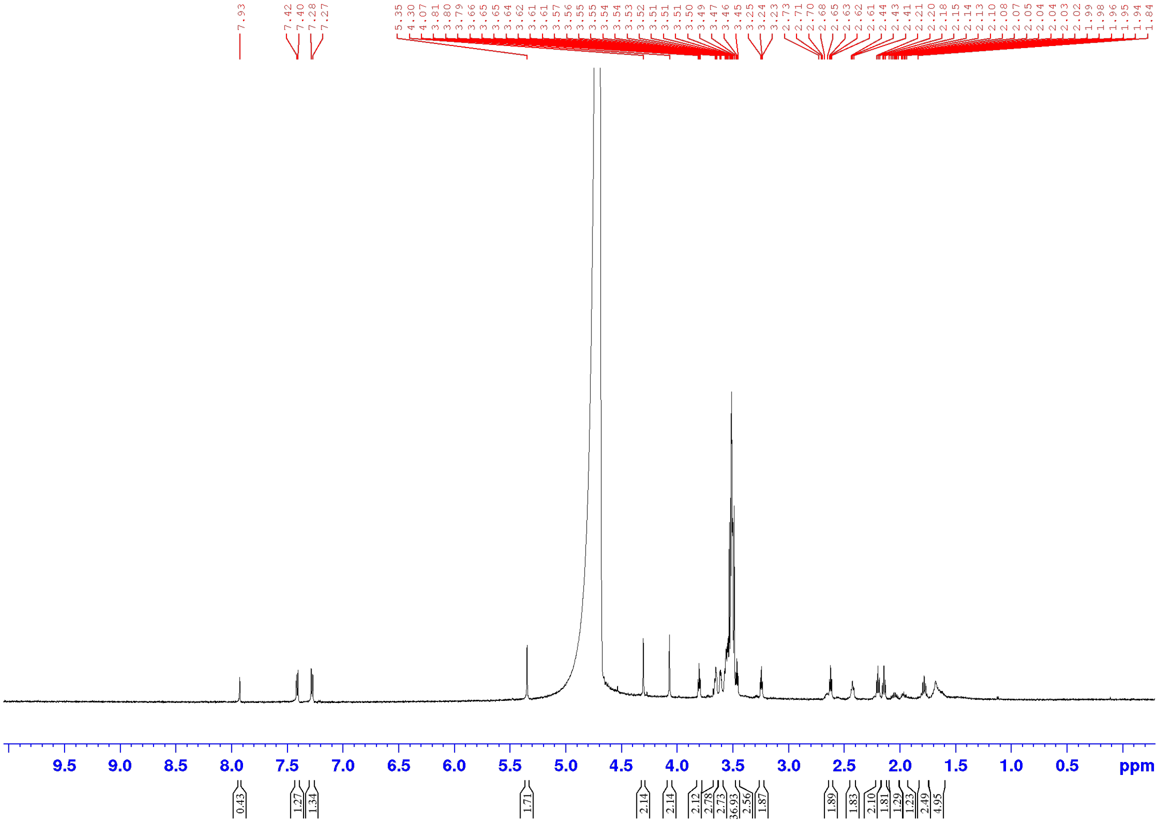

3.7. (2*S*,4*S*)-2-Amino-4-(4-((4-((*E*)-(4-(1-(4-(((2-amino-9*H*-purin-6-yl)oxy)methyl)phenyl)-3,7,47-trioxo-11,14,17,20,23,26,29,32,35,38,41,44-dodecaoxa-2,8,48-triazapentacontan-50-amido)phenyl)diazenyl)phenyl)amino)-4-oxobutyl)pentanedioic acid (BGAG$_{12}$-v2-h8)

^{1}H-NMR

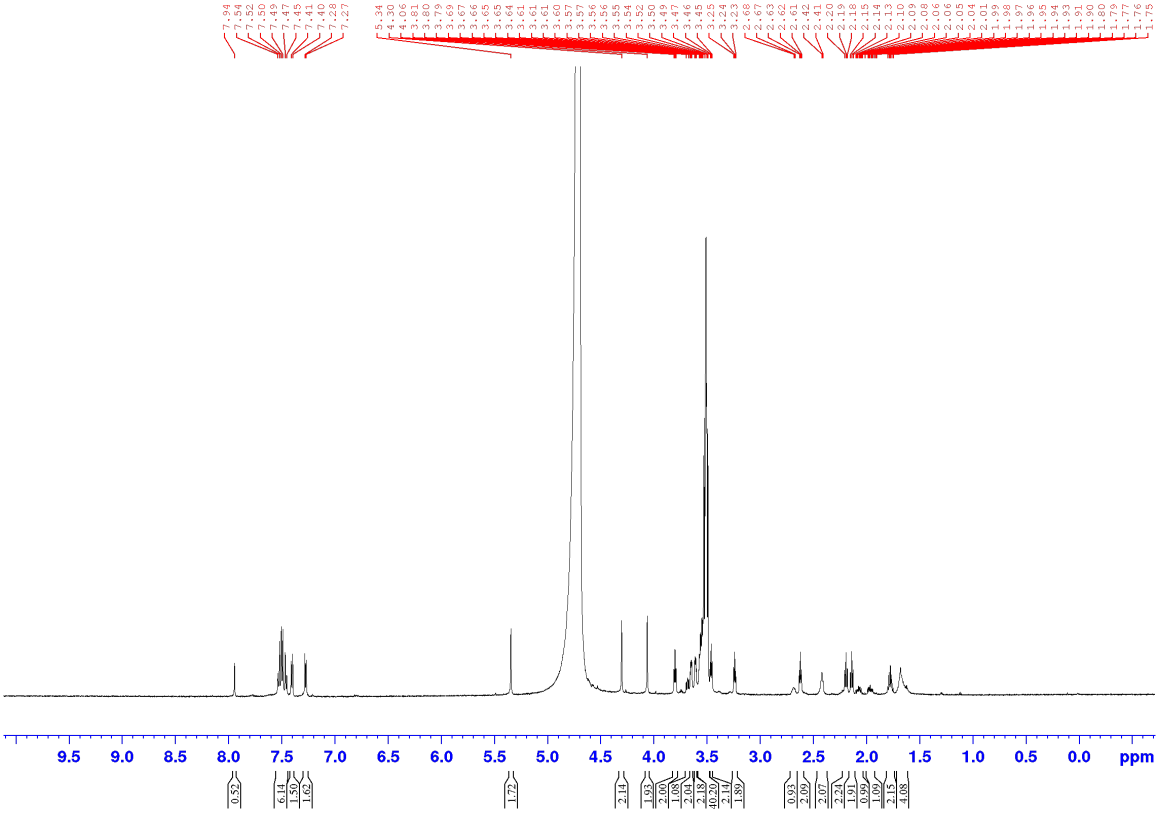

3.8. Overlay of ^{1}H NMR spectra of BGAG$_{12}$-v2-h8 (blue) and BGAG$_{12}$-v2-d8 (red)

^{1}H-NMR

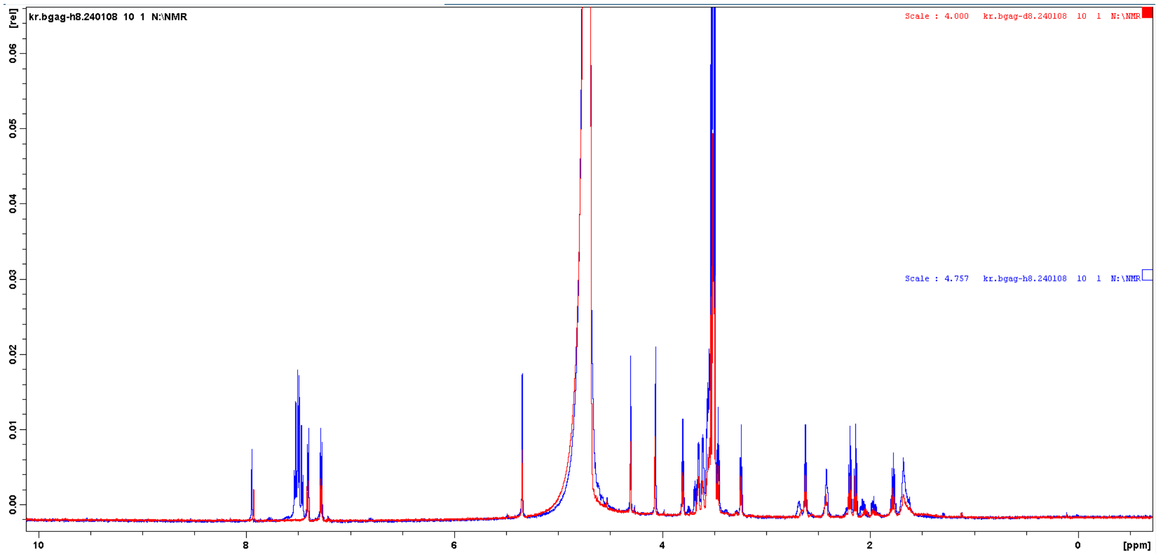

4. DFT Calculations

All (density functional theory) calculations were performed using the TURBOMOLE program package, version 7.7.1.[3] Structure optimizations and IR Spectra computations were performed at the PBE0[4,5]/def2-TZVPP[6] level of theory using the identity of the resolution (RI) approximation for the Coulomb contribution in conjunction with standard TURBOMOLE auxiliary basis sets for all nuclei (def2-universal).[7] The TURBOMOLE internal grid setting of size "3" was employed throughout.[8] Convergence criteria regarding the energy changes were set to 10^{-8} Hartree, while additionally enforcing convergence of changes in the density-matrix of 10^{-7} a.u.; Convergence of the optimized structures employed gradient norms $<=10^{-5}$ a.u.. The conductor-like screening model (COSMO) was applied to mimic the DMSO solvent environment using a dielectric constant (ε) of 46.7.[9]

Harmonic vibrational spectra were computed numerically using the NumForce subroutine employing displacements of 0.02 a.u.. Derivatives of the quadrature weights were additionally set to improve the gradients of the displaced structures in the optimizations and calculation of vibrational data. The resulting spectral data was transformed using the Gallier tool, as provided by TURBOMOLE, using Gaussian broadening with a full width at half maximum of 4 cm^{-1}.

Table S1: Wavenumbers and IR intensities for *trans*-AB-h10 and *trans*-AB-d10

trans-AB-h10: $C_{12}N_2H_{10}$			*trans*-AB-d10: $C_{12}N_2D_{10}$			
wavenumber in cm^{-1}	IR intensity		wave number in cm^{-1}	IR intensity		
528.2	52.1		508.79	42.7		CN Bend
546.28	7.5		530.47	10.3		CC Bend
566.26	32.4		489.05	77.8		CC Bend (out plane) // C-N-H Flapping/Bend
711.72	171.3		563.43	70.5		CH Bend (out Plane)
813.31	105.0		681.07	10.6		CH Bend (out Plane)
972.21	16.3		821.85	8.3		CH Bend (out Plane)
1048.07	28.6		777.01	9.3		CH Bend (in plane)
1101.86	28.1		821.95	17.8		CH Bend (in plane)
1172.29	52.1		834.67	44.7		CH Bend (in plane)
1274.4	58.5		1214.34	38.6		CN Stretch (CH Bend involved)
1334.32	2.5		1054.35	6.9		Ring turn /CH Bend
1375.75	22.8		1363.18	32.1		CC stretch
1493.83	18.5		1379.94	9.0		CC stretch (H bend involved)
1527.46	13.3		1424.09	0.2		CC stretch (H bend involved)
1644.53	10.5		1609.16	11.2		CC stretch (H bend involved)
1659.41	16.2		1627.13	21.3		CC stretch
3200	14.0		2363.44	8.1		CH Stretch Mode
3207.44	32.0		2372.81	14.3		CH Stretch Mode
3216.6	61.2		2384.24	18.8		CH Stretch Mode
3226.03	15.4		2389.36	28.9		CH Stretch Mode

Table S2: Wavenumbers and IR intensities for *cis*-AB-h10 and *cis*-AB-d10

cis-AB-h10: $C_{12}N_2H_{10}$			*cis*-AB-d10: $C_{12}N_2D_{10}$			
wave number in cm^{-1}	IR intensity		wave number in cm^{-1}	IR intensity		
292.91	11.9		280.13	12.2		CN Bend
441.3	5.4		410.72	9.6		CN Bend
510.77	10.4		480.18	39.6		CN Bend
711.3	72.2		554.34	103.2		CH Bend (out Plane)
713.2	48.5		557.24	27.8		CH Bend (out Plane)
718.18	116.0		506.25	10.9		CH Bend (out Plane) / CN involved
786.91	79.8					CH Bend (out Plane)
803.57	24.9					CH Bend (out Plane)
			906.68	20.7		CN Bend (H involved)
955.6	42.1					CH Bend (out Plane)
1106.72	27.5		829.8	15.1		CH Bend (in plane)
1519.35	16.2		1397.51	5.4		CC stretch (H bend involved)
1633.85	35.7		1607.03	7.9		NN/CC Stretch (H bend involved)
1639.39	10.0		1603.53	5.8		CC stretch (H bend involved)
1672.77	47.8		1663.28	72.5		NN/CC Stretch (H bend involved)
3198.4	10.5		2361.67	6.5		CH Stretch Mode
3206.18	19.9		2371.71	10.8		CH Stretch Mode
3206.29	11.6		2371.84	3.2		CH Stretch Mode
3213.98	42.5		2380.46	19.2		CH Stretch Mode
3218.96	12.0		2387.57	12.4		CH Stretch Mode
3219.03	10.7		2387.73	7.4		CH Stretch Mode

5. Photochemical actinometry

The ferrioxalate method was used to calculate the photon flux using the protocol described in (Stranius and Börjesson, 2017). In short, a 2 ml (V_1) solution of ferrioxalate (30 mM in 0.2 N H_2SO_4) was irradiated (using a 450 W Xe lamp connected with a monochromator, center wavelength 385 nm or 365 nm, slit size 5 nm) under stirring. 0.5 ml (V_2) of the irradiated volume together with buffer (1.2 M NaOAc + 0.72 N H_2SO_4, 1 ml), and phenanthroline (6 mM, 2 ml) was diluted to 25 ml (V_3) and left to react for 1 h, where after the absorbance of the tris-phenanthroline complex was recorded at 510 nm (ε_{510nm}=11,100 M^{-1} cm^{-1}). The photon flux was then calculated using:

$$I = slope * \frac{V_1 * V_2 * 10^{-3}}{V_3 * \varepsilon_{510nm} * l * \varphi} \qquad (1)$$

Where the linear fit in Figure S1 was used to get the slope, l (small L) is the path length of the cuvette, and ϕ is the photochemical quantum yield of the process (~1.18 @ 385 nm, and ~1.21 @ 365 nm). Using equation 1 the photon flux was determined to be $3.3*10^{15}$ photon/s @ 385 nm, and $5.3*10^{15}$ photon/s @ 365 nm.

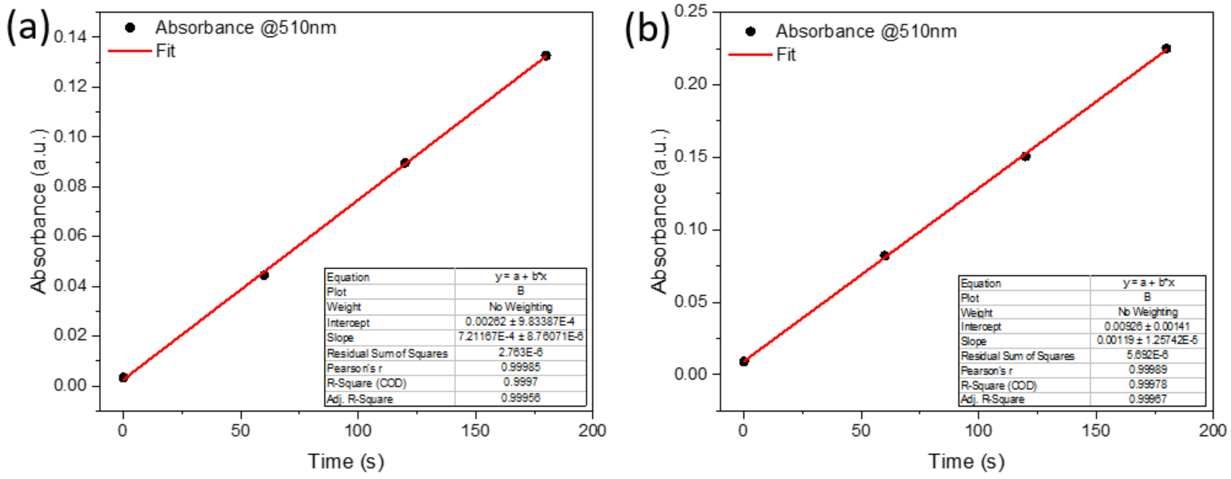

Figure S1: Absorbance of the tris-phenanthroline iron (II) complex as a function of irradiation time of potassium ferrioxalate (black squares). The red line shows a linear fit, which is used to calculate the photon flux; with the Irradiation wavelength (a) 385 nm, and (b).365 nm.

6. Photoisomerization quantum yield measurements

The rate of a photoisomerization event from species A (*trans*) to B (*cis*) can in the absence of any thermal backreaction be described with (Stranius and Börjesson, 2017)[10]:

$$\frac{dA}{dt} = -\frac{\varphi_A \cdot I \cdot \beta_A(t)}{N_A \cdot V} + \frac{\varphi_B \cdot I \cdot \beta_B(t)}{N_A \cdot V} \quad (2)$$

Where N_A is Avogadro's number, and V the volume of the sample (in dm^3). β describes the fraction of light absorbed by each species:

$$\beta_A(t) = \frac{[A]\cdot\varepsilon_A}{[A]\cdot\varepsilon_A + [B]\cdot\varepsilon_B}(1 - 10^{-Abs(t)}) \quad (3)$$

Where Abs is the absorbance of the sample at the wavelength of irradiation, and ε is the molar absorptivity of each species (AQ-h8-trans 11,700 M^{-1} cm^{-1}, AQ-h8-cis 584 M^{-1} cm^{-1}, AQ-d8-trans 11,000 M^{-1} cm^{-1}, AQ-d8-cis 536 M^{-1} cm^{-1}). Equation 2 was used to fit experimental data using the ϕ as fitting parameters. The *trans* to *cis* quantum yield for AQ-h8 and AQ-d8 was determined to be 0.38 ± 0.011 and 0.43 ± 0.008, respectively with errors indicating a 95% confidence interval. Experiments was performed in triplicates to acquire statistics.

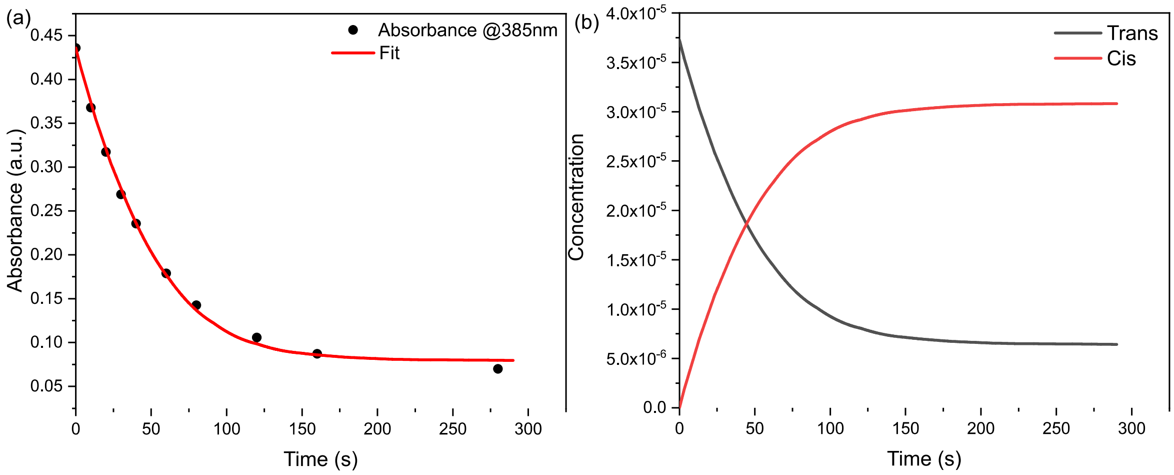

Figure S2: a) Absorption of a mixture of *cis* and *trans* of AQ-h8 in PBS buffer as a function of time when simultaneously irradiating the solution and a fit to equation 2, giving the quantum yield of photoisomerization. b) Concentration of the *trans* and *cis* isomers of AQ-h8 during the photoisomerization event.

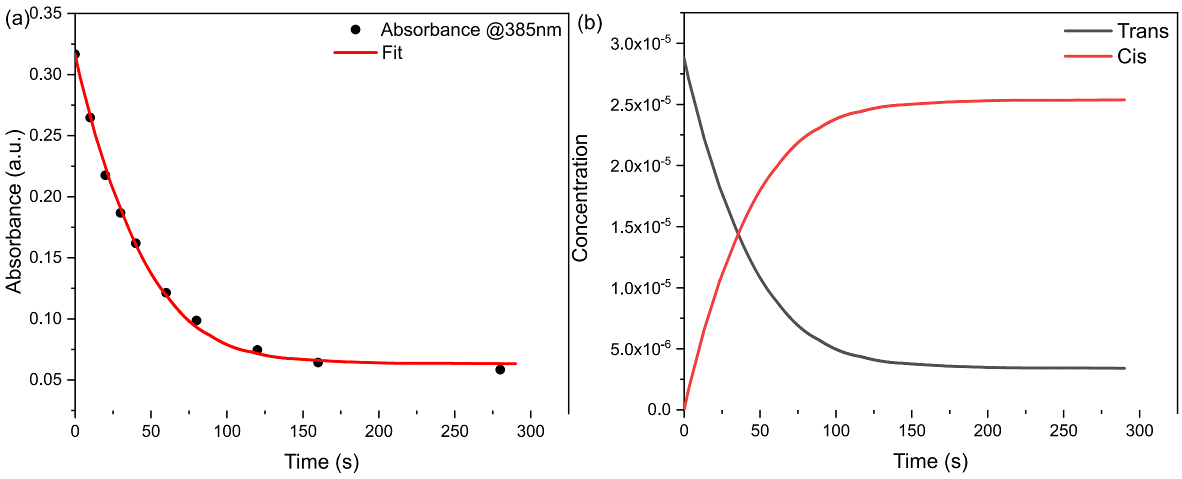

Figure S3: a) Absorption of a mixture of *cis* and *trans* of AQ-d8 in PBS buffer as a function of time when simultaneously irradiating the solution and a fit to equation 2, giving the quantum yield of photoisomerization. b) Concentration of the *trans* and *cis* isomers of AQ-d8 during the photoisomerization event.

The photoisomerization quantum yield of AB-h10, and AB-d10 were determined using 365 nm irradiation. The molar absorptivity of each species were set to (AB-h10-trans 1860 M^{-1} cm^{-1}, AB-h10-cis 81 M^{-1} cm^{-1}, AB-d10-trans 2020 M^{-1} cm^{-1}, AQ-d10-cis 99 M^{-1} cm^{-1}). Equation 2 was used to fit experimental data using the ϕ as fitting parameter. The *trans* to *cis* quantum yield for AB-h10 and AB-d10 was determined to be 0.14 ± 0.026 and 0.15 ± 0.016, respectively with errors indicating a 95% confidence interval.

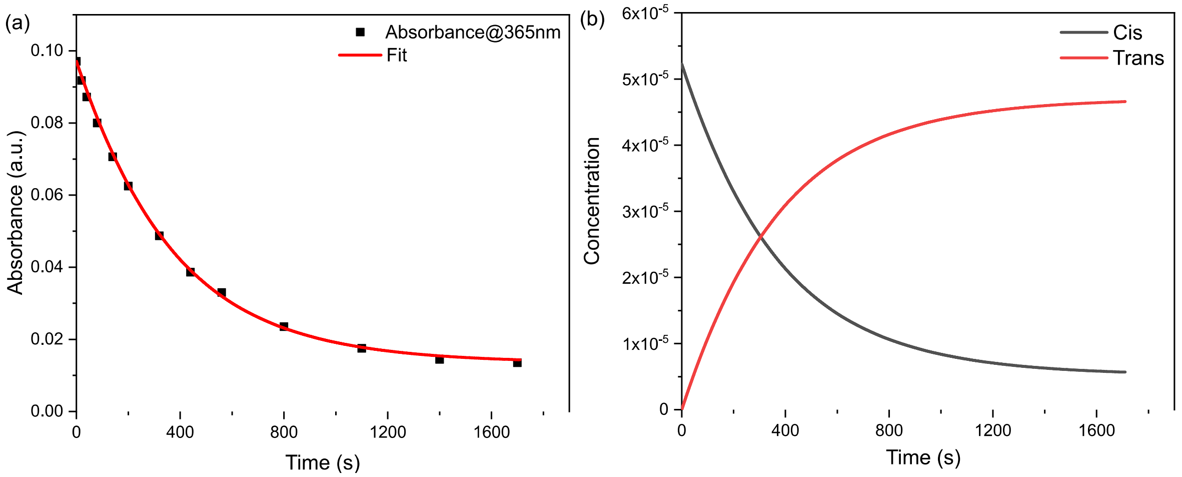

Figure S4: a) Absorption of a mixture of *cis* and *trans* AB-h10 in DMSO as a function of time when simultaneously irradiating the solution and a fit to equation 2, giving the quantum yield of photoisomerization. b) Concentration of the *trans* and *cis* isomers of AB-h10 during the photoisomerization event.

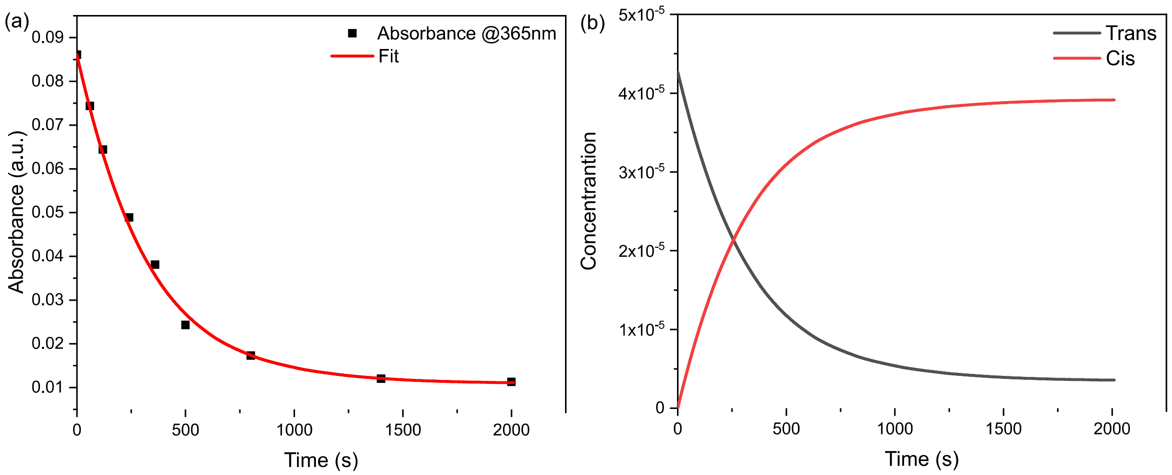

Figure S5: a) Absorption of a mixture of *cis* and *trans* AB-d10 in DMSO as a function of time when simultaneously irradiating the solution and a fit to equation 2, giving the quantum yield of photoisomerization. b) Concentration of the *trans* and *cis* isomers of AB-d10 during the photoisomerization event.

Table S3: Photoisomerization quantum yield of AQ-h8 and AQ-d8 for three different experiments.

Experiment	Photoisomerization QY of AQ-h8	Photoisomerization QY of AQ-d8
1	0.3711	0.4276
2	0.3821	0.4329
3	0.3757	0.4245
Average	0.38	0.43

Table S4: Photoisomerization quantum yield of AB-h10 and AB-d10 for three different experiments.

Experiment	Photoisomerization QY of AB-h10	Photoisomerization QY of AB-d10
1	0.1504	0.1473
2	0.1378	0.1562
3	0.1246	0.1405
Average	0.14	0.15

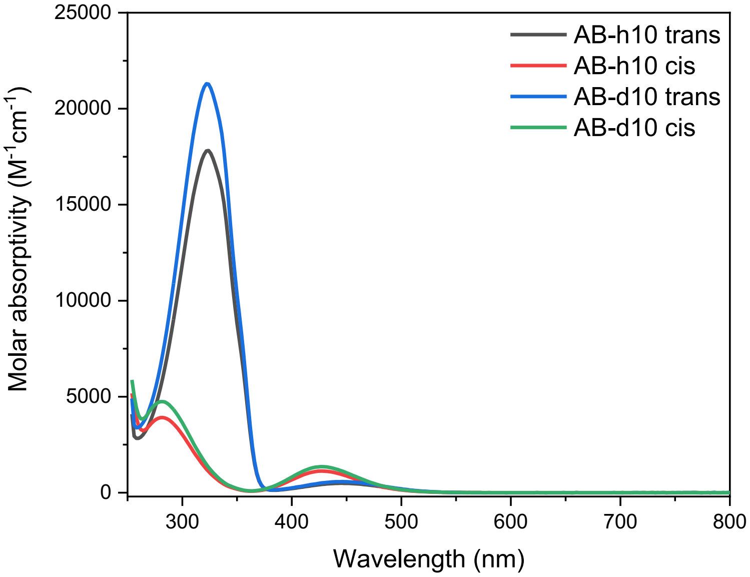

Figure S6: Molar absorptivity spectra of *cis* and *trans* isomers of both AB-h10 and AB-d10. The molar absorptivity of the *trans* form was directly measured, and the molar absorptivity of the *cis* form was constructed by taking an absorption spectra of the *cis* form and scaling it to the value of the molar absorptivity of the *trans* form at the isosbestic point.

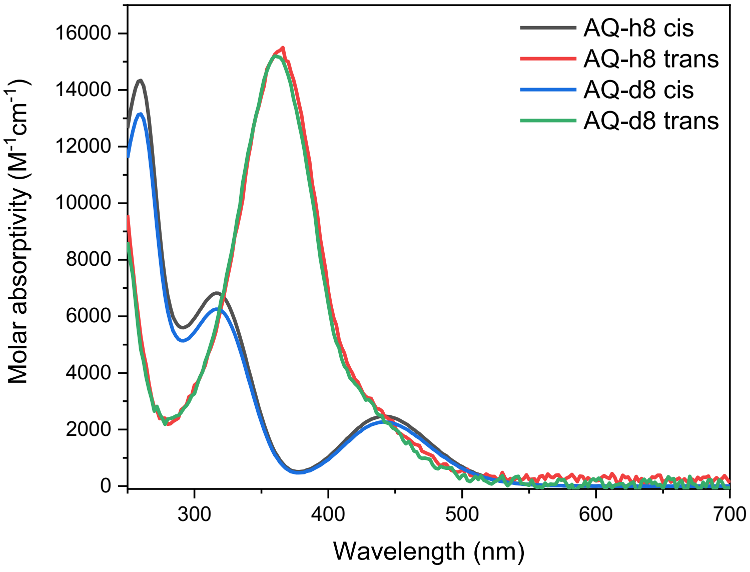

Figure S7: Molar absorptivity spectra of *cis* and *trans* isomers of both AQ-h8 and AQ-d8. The molar absorptivity of the *trans* form was directly measured, and the molar absorptivity of the *cis* form was constructed by taking an absorption spectra of the *cis* form and scaling it to the value of the molar absorptivity of the *trans* form at the isosbestic point.

For the photoisomerization from *cis* to *trans*, the yield can be directly extracted the fits in Figures Q2-5. However, a more accurate methods is to excite at an isosbestic point. When exciting at an isosbestic point and after reaching the photostationary state, Equation 2 reduces to:

$$\frac{\varphi_A}{\varphi_B} = \frac{[B]}{[A]} \qquad (4)$$

The benefit of using Equation 4 is that the molar absorptivity at the excitation wavelength is not needed in the analysis. Only the photoisomerization quantum yield of the forward rection together with the molar ratio of the two species are needed. The drawback of this method is that the photoisomerization reaction typically is not determined at the isosbestic point, the analysis therefore assumes that Khasha's law is valid (that the photoisomerization yield does not depend on excitation wavelength). All molecules were irradiated at an isosbestic point, and the wavelength of the absorbance maximum of the *trans* isomer were used to assess the concentration at time zero and at the photostationary state.

Table S5: The absorbance was evaluated at 360 nm and 322 nm for AQ-h/d8 and AB-h/d10, respectively. A_0 and A_{PSS} are the total absorbance of the solution at the absorption maxima (360/322 nm) without irradiation and at the photostationary state. ε_{cis}, and ε_{trans} are the absorption coefficients for the *cis* and *trans* isomer, respectively.

	I_{ex} (nm)	Abs_0	Abs_{PSS}	ε_{trans}	ε_{cis}	$QY_{cis\text{-}trans}$
AQ-h8	442	0.6686	0.4445	15257	1210	0.66
AQ-d8	442	0.5002	0.3441	15200	1110	0.84
AB-h10	268	1.2896	1.0752	17800	1230	0.64
AB-d10	268	0.9891	0.8002	21300	1480	0.57

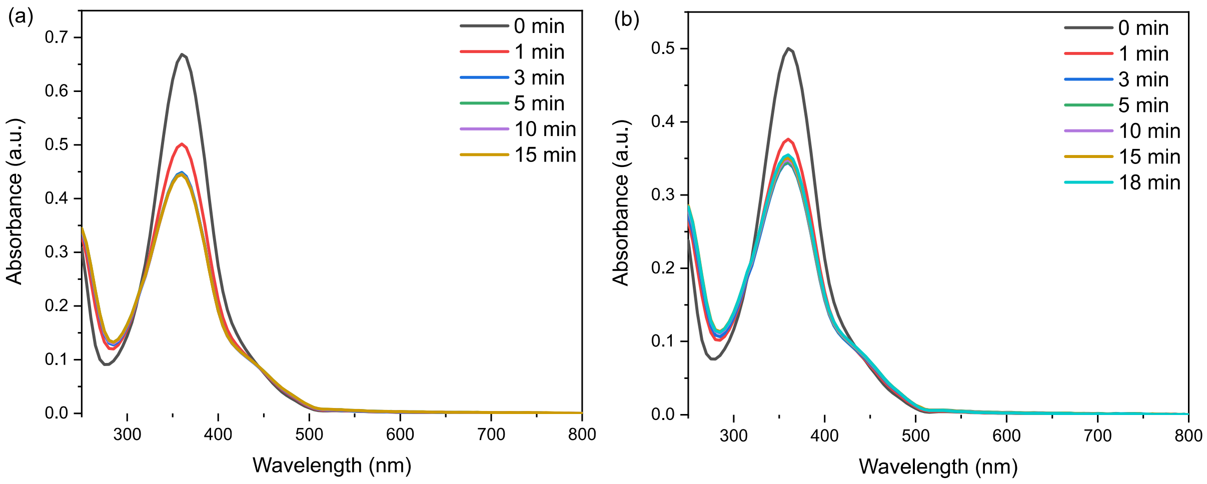

Figure S8: Absorption spectra of AQ-h8 (a), and AQ-d8 (b) in PBS buffer when irradiating with 442 nm light for 0 to 18 minutes.

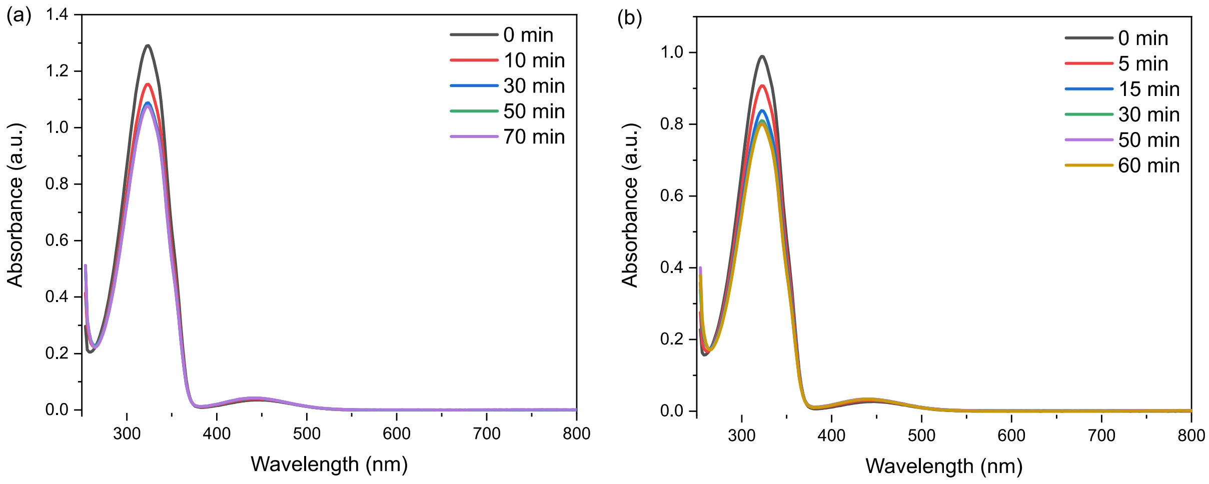

Figure S9: Absorption spectra of AB-h10 (a), and AB-d10 (b) in DMSO with irradiating with 268 nm light for 0 to 70 minutes.

7. Supplementary Figures

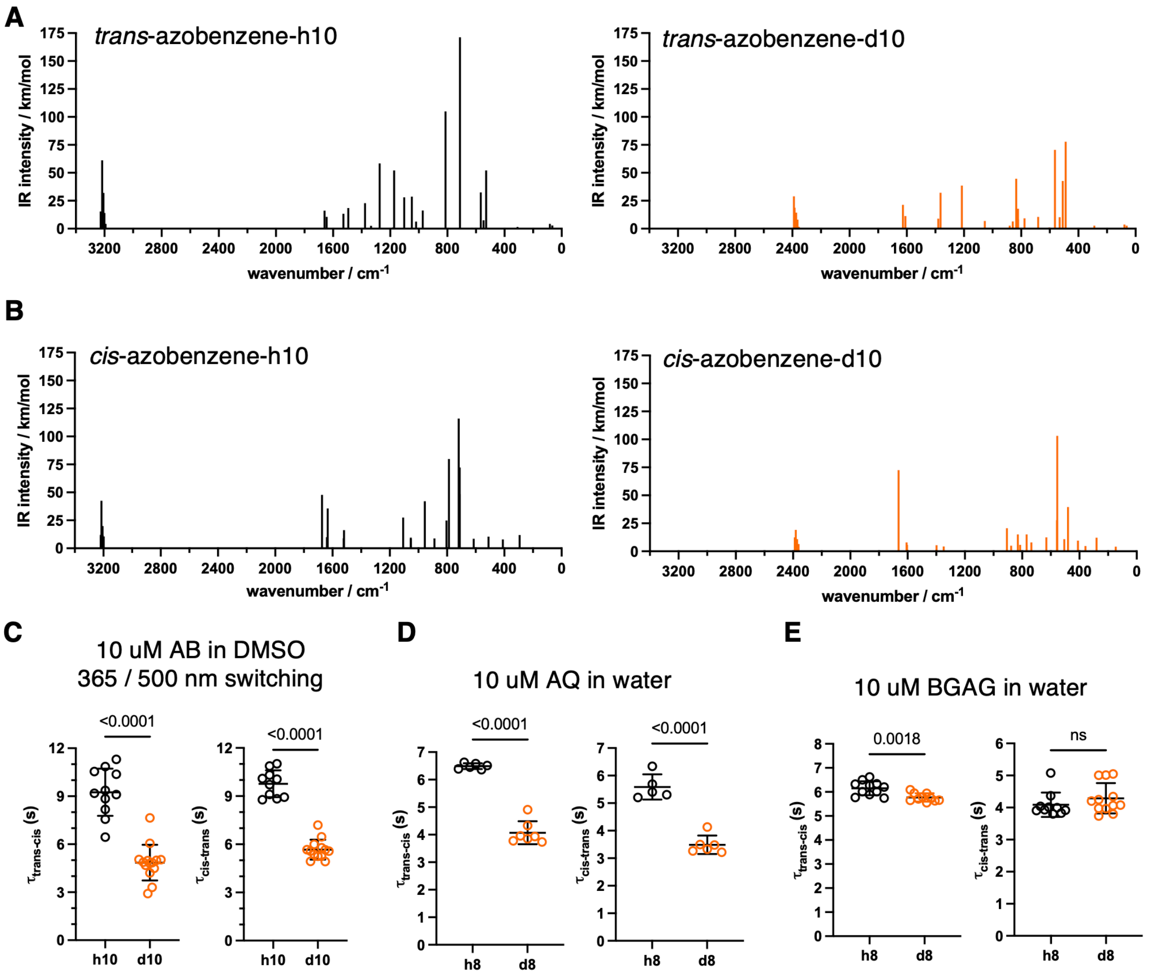

Figure S10: DFT calculated IR spectra and photoswitching of AB-h10 and AB-d10. **A)** Individually plotted IR spectra of *trans*-AB-h10 and *trans*-AB-d10. **B)** Individually plotted IR spectra of *cis*-AB-h10 and *cis*-AB-d10. **C)** Photoswitching of AB-h10 and AB-d10 confirms kinetic trends even under different irradiation wavelength and intensity and at 10 uM in DMSO. **D**) As for C, but with AQ in water. **E**) As in D), but with BGAG.

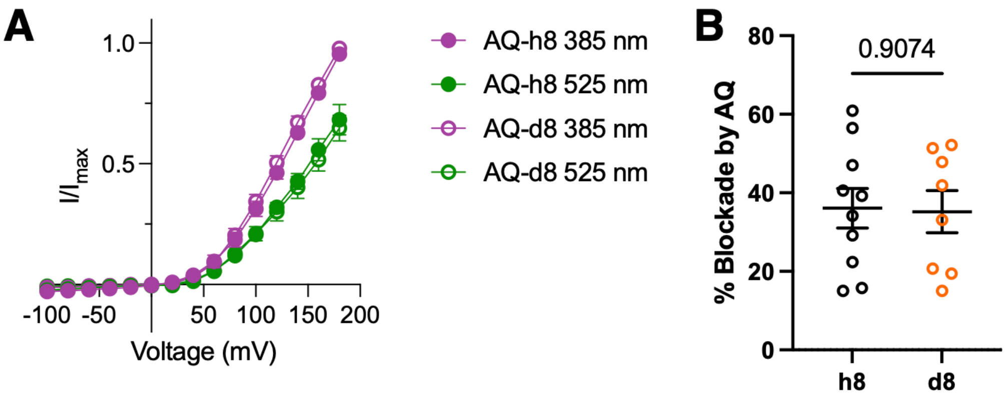

Figure S11: BK channel blockade by AQ-h8 or AQ-d8 is comparable. **A)** Current-voltage relationship for BK channel activation in response to a step pulse protocol showing effect of trans-induced photo-block. **B)** Quantification of the % block for each compound calculated as 100(1-x) with x being the difference between *cis* and *trans* total current measured at +180 mV for each individual cell. P-value of unpaired t-test is reported in panel B.

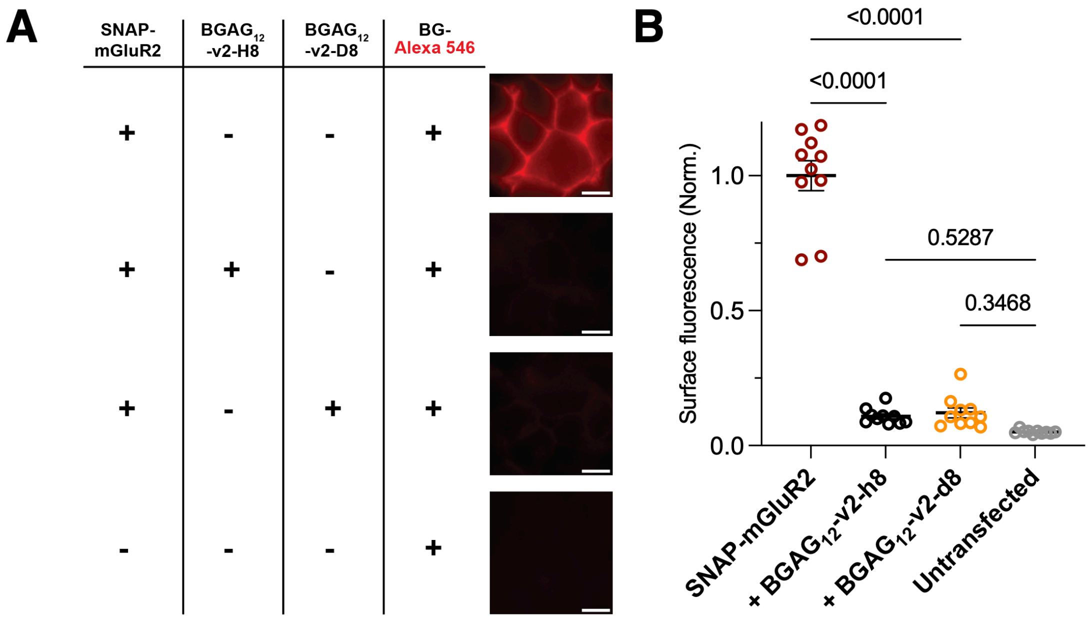

Figure S12: BGAG photoswitching of SNAP-mGluR2. **A-B)** Labelling efficiency of the BGAG compounds. We tested the labelling efficiency of the SNAP-tag by the BGAG compounds using a pulsed-chase competition assay. P-values of One-way ANOVA with Tukey's multiple comparisons test are reported in panel C.

8. Supplementary Schemes

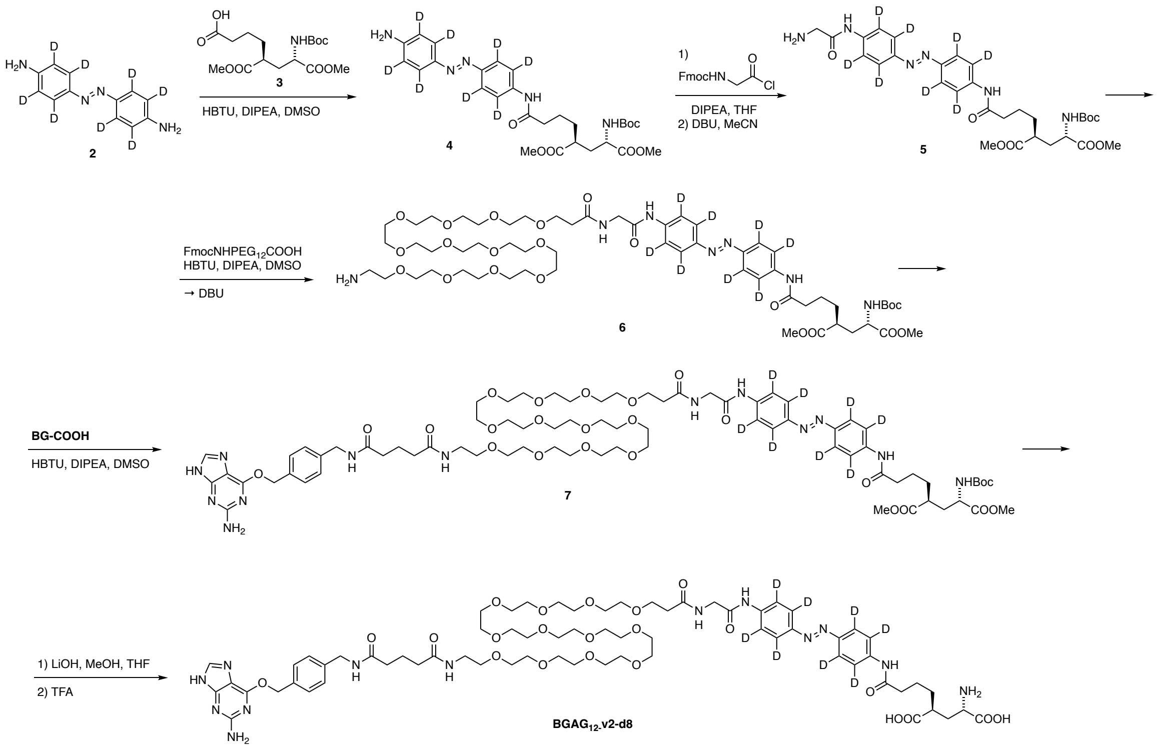

Scheme S1: Chemical synthesis of BGAG$_{12}$-v2-d8.

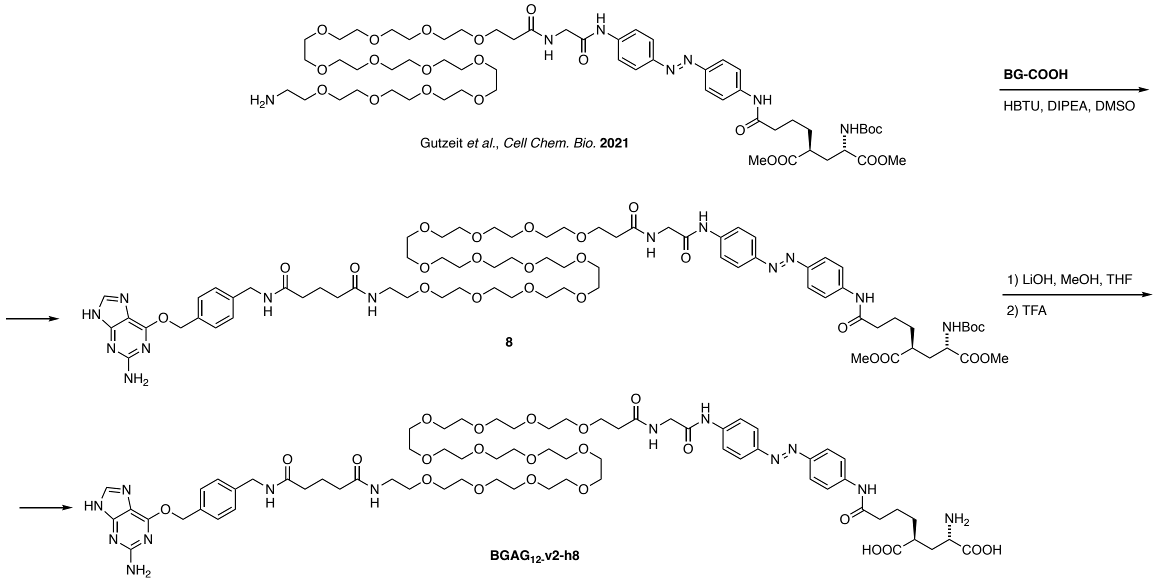

Scheme S2: Chemical synthesis of BGAG$_{12}$-v2-h8.

9. Cell culture, molecular biology and patch clamp electrophysiology

HEK293 cells were cultured in Dulbecco's Modified Eagle Medium (DMEM; Corning) supplemented with 10% fetal bovine serum (FBS) and maintained at 37 ºC and 5% CO_2. Cells were seeded at low density in poly-L-lysine coated 18 mm coverslips and transfected the following day with Lipofectamine 2000 (Thermo Fisher Scientific). Plasmid expressing BK channel human alpha subunit (pBNJ13-hSlo)[11] was kindly gifted by Prof. Teresa Giraldez (University of La Laguna, Spain). This construct was used for testing AQ compounds. For BGAG recordings, SNAP-mGluR2[12], GIRK1-F137S[13] and tdTomato as a transfection marker were co-transfected in cells in a 1:1:0.2 ratio.

Whole cell patch clamp recordings were performed 24 hr after transfection using an Axopatch 200B amplifier and a Digidata 1550B interface controlled by pClampex software (Molecular Devices). Recordings were performed in a bath solution containing (in mM): 120 KCl, 25 NaCl, 10 HEPES, 2 $CaCl_2$, 1 $MgCl_2$. Pipettes of 3-5 MΩ resistance were filled with intracellular solution (in mM: 140 KCl, 10 HEPES, 5 EGTA, 3 $MgCl_2$, 3 Na_2ATP, 0.2 Na_2GTP). For AQ compounds, AQ-h8 and AQ-d8 were added to a final concentration of 1 mM in the pipette solution. For BGAG, cells were labelled with 1 μM of BGAG12-v2-h8 or BGAG12-v2-d8 for 45 min at 37 ºC in extracellular solution. Labeling efficiency was measured using a fluorophore competition assay as previously.[14] Photoactivation of the compounds was obtained through a computer controlled CoolLED pE-4000 attached to an inverted microscope and through a 40x objective. Light intensities at the focal plane were (in mW/mm^2): 5.6 for 385 nm and 4.9 for 525 nm. For AQ compound photoswitching, a 0.1% neutral density ND filter (Chroma) was added to the 385 nm illumination path to produce lower light conditions for kinetics analysis (5.57 $\mu W/mm^2$).

To obtain an I-V curve for BK channel activation, a step protocol of 50 ms of pulse ranging from - 100 mV to +200 mV in +20 mV increments was recorded. This was done in the presence of either wavelength (385 nm or 525 nm) throughout each sweep. Steady-state current at the end of the pulse, normalized to the maximum current observed in each individual cell, was plotted against the voltage applied to each step. The protocol for photoswitching of AQ compounds consisted of a voltage clamp of the cell at +60 mV and applying pulses of 20 s of 385 nm immediately followed by 10 s of 525 nm light. For BGAG recordings, photoactivation by 385 nm was performed until the mGluR2 evoked GIRK current was in a steady state and after that, was quickly switched off by 525 nm light. Photoswitch efficiency was calculated as the amplitude of the 385 nm evoked current divided by the amplitude of the current response to saturating 1 mM glutamate.

All cellular data comes from at least three separate transfections/experimental days. Data was analyzed using Clampfit (Molecular Devices) and Prism 9 (GraphPad). AQ and BGAG *trans*-to-*cis* kinetics were quantified by fitting the evoked currents to a single exponential.

10. References

1. Banghart, M. R. *et al.* Photochromic blockers of voltage-gated potassium channels. *Angew. Chem. Int. Ed. Engl.* **48**, 9097–9101 (2009).
2. Gutzeit, V. A. *et al.* A fine-tuned azobenzene for enhanced photopharmacology in vivo. *Cell Chemical Biology* **28**, 1648-1663.e16 (2021).

3. TURBOMOLE V7.7.1 2023, a development of University of Karlsruhe and Forschungszentrum Karlsruhe GmbH, 1989–2007, TURBOMOLE GmbH since 2007; available from https://www.turbomole.org.
4. Adamo, C. & Barone, V. Toward chemical accuracy in the computation of NMR shieldings: the PBE0 model. *Chemical Physics Letters* **298**, 113–119 (1998).
5. Adamo, C. & Barone, V. Toward reliable density functional methods without adjustable parameters: The PBE0 model. *The Journal of Chemical Physics* **110**, 6158–6170 (1999).
6. Weigend, F. & Ahlrichs, R. Balanced basis sets of split valence, triple zeta valence and quadruple zeta valence quality for H to Rn: Design and assessment of accuracy. *Phys. Chem. Chem. Phys.* **7**, 3297 (2005).
7. Weigend, F. Accurate Coulomb-fitting basis sets for H to Rn. *Phys. Chem. Chem. Phys.* **8**, 1057 (2006).
8. Treutler, O. & Ahlrichs, R. Efficient molecular numerical integration schemes. *The Journal of Chemical Physics* **102**, 346–354 (1995).
9. Klamt, A. & Schüürmann, G. COSMO: a new approach to dielectric screening in solvents with explicit expressions for the screening energy and its gradient. *J. Chem. Soc., Perkin Trans. 2* 799–805 (1993) doi:10.1039/P29930000799.
10. Stranius, K. & Börjesson, K. Determining the Photoisomerization Quantum Yield of Photoswitchable Molecules in Solution and in the Solid State. *Sci Rep* **7**, 41145 (2017).
11. Giraldez, T., Hughes, T. E. & Sigworth, F. J. Generation of Functional Fluorescent BK Channels by Random Insertion of GFP Variants. *The Journal of General Physiology* **126**, 429–438 (2005).
12. Doumazane, E. *et al.* A new approach to analyze cell surface protein complexes reveals specific heterodimeric metabotropic glutamate receptors. *FASEB journal : official publication of the Federation of American Societies for Experimental Biology* **25**, 66–77 (2011).

13. Vivaudou, M. *et al.* Probing the G-protein Regulation of GIRK1 and GIRK4, the Two Subunits of the KACh Channel, Using Functional Homomeric Mutants. *Journal of Biological Chemistry* **272**, 31553–31560 (1997).

14. Levitz, J. *et al.* Dual optical control and mechanistic insights into photoswitchable group II and III metabotropic glutamate receptors. *Proc. Natl. Acad. Sci. USA* **114**, E3546–E3554 (2017).
